# Health co-benefits and trade-offs of carbon pricing: a narrative synthesis

**DOI:** 10.1080/14693062.2024.2356822

**Published:** 2024-06-02

**Authors:** Soledad Cuevas, Daniel Nachtigall, Aimee Aguilar Jaber, Kristine Belesova, Jane Falconer, Andy Haines, Tamzin Reynolds, Tobias Magnus Schuster, Sarah Whitmee, Rosemary Green

**Affiliations:** a London School of Hygiene and Tropical Medicine, London, UK; b Instituto de Economía, Geografía y Demografía (IEGD), Consejo Superior de Investigaciones Científicas (CSIC), UK; c OECD, Paris, France; d Imperial College London, London, UK

**Keywords:** Carbon pricing, health co-benefits, mitigation, policy analysis, evidence synthesis

## Abstract

Carbon pricing is a key component of current climate policy agendas. There are a variety of societal and health impacts from carbon pricing interventions (e.g. from improved air quality). A better understanding of potential health impacts and how they depend on context and policy design is crucial to improve the political feasibility and fairness of carbon pricing. Recent reviews have synthesized evidence on the effectiveness, equity and perceptions of carbon pricing and on the health co-benefits of mitigation. This review provides a narrative structured synthesis of the health impacts of carbon pricing. We identified 58 relevant publications of which all were modelling studies. We classify review findings into policy-relevant categories, synthesizing information on how carbon pricing affects health outcomes when implemented in different contexts, in isolation or as part of policy mixes. Findings suggest that internalization of health co-benefits in optimal price level estimates could lead to substantial mitigation in some regions. There are also opportunities to design carbon pricing to improve health outcomes, including through progressive or targeted use of revenues to improve food security, subsidize healthier diets or promote active transportation. Revenue use, price differentiation, market size and permit allocation of emissions trading schemes (ETS), and interaction with other public health or mitigation policies all influence health outcomes. Overall, the health impacts of carbon pricing are highly context-specific and further evidence is needed, particularly on health inequalities and ex-post analysis. However, existing evidence suggests that it is possible to design health-beneficial carbon pricing policies, thus enhancing policy acceptability and feasibility.

## Key policy insights

- Internalizing health co-benefits into optimal carbon pricing estimates could lead to substantial emission reductions in some regions, although additional action is needed to encourage price levels compatible with climate targets
- Health co-benefits can be boosted through key elements of policy design including price differentiation across commodities and regions, revenue use or ETS design
- Careful policy design can enhance health gains at a small or insignificant cost in terms of climate mitigation effectiveness and efficiency.
- The implementation of carbon pricing as part of broader policy mixes with a strong focus on progressivity and equity is crucial to achieving health co-benefits

## Introduction

Carbon pricing (CP) can be a cost-effective intervention to reduce GHG emissions, while also having potentially important health co-benefits, improving air quality, encouraging active transportation, redistributing wealth or raising funds for healthcare and other health-related public goods (Cuevas and Haines 2016). Health co-benefits are the positive impacts on human health that can result from carbon abatement interventions, but which are not the result of climate change mitigation itself. At the same time, there is also the potential for carbon pricing mechanisms to lead to negative health and wellbeing impacts, especially if socioeconomic inequalities are exacerbated (Parry et al., 2015).

**Figure 1.**
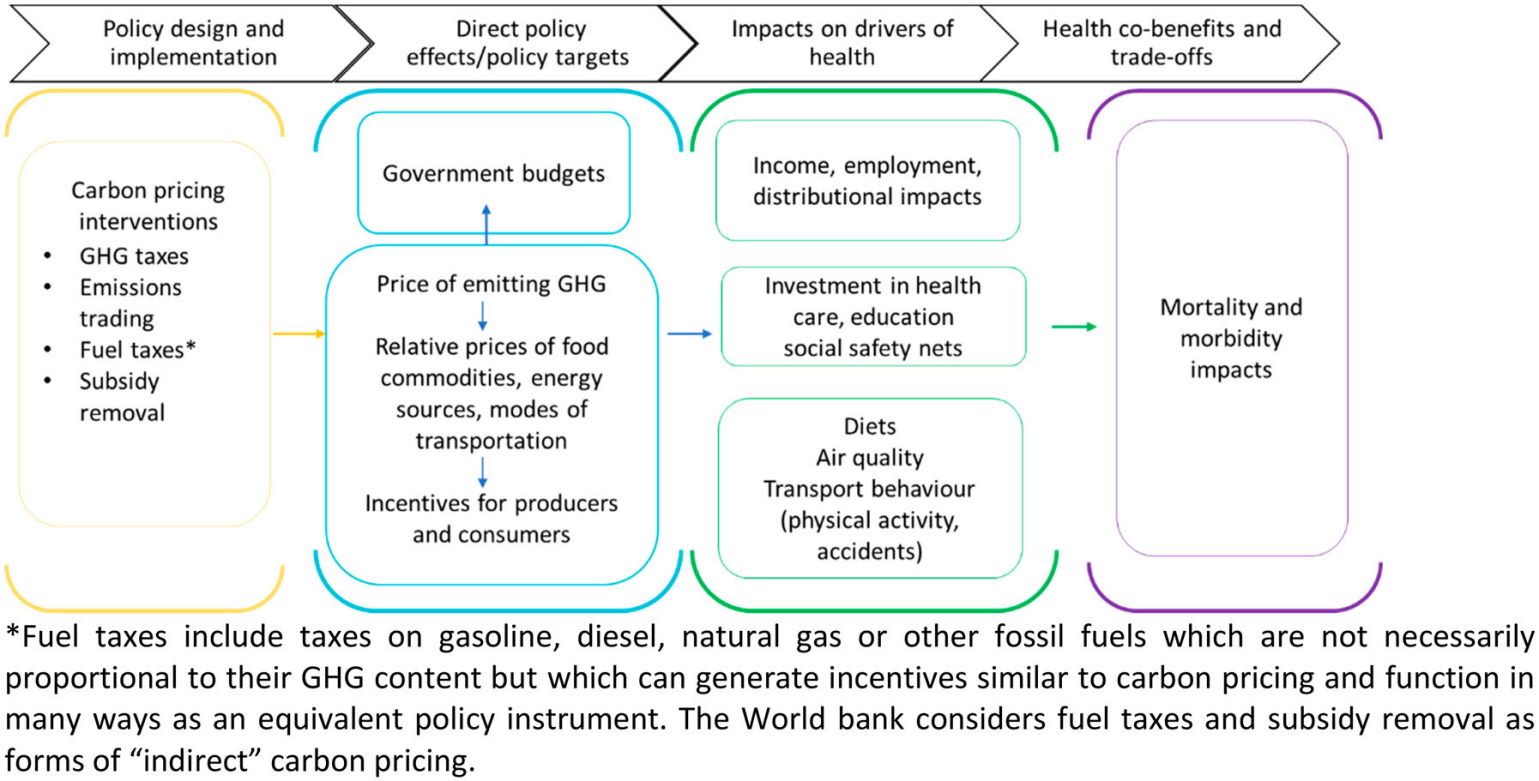
Carbon pricing and health.

Understanding the importance of health as well as other societal impacts of CP is crucial in order to design and implement mitigation policies that can achieve their emissions reduction goals while supporting human well-being and equity (See Figure 1). An improved understanding of health co-benefits can also potentially increase societal and policy support (Dasandi et al., 2022; Nemet et al., 2010) which has been identified as a key concern for the success of carbon pricing interventions (Klenert et al., 2018).

In recent years it has been argued by many that CP interventions cannot, on their own, drive the necessary changes to achieve deep decarbonization throughout society and meet climate targets (Green, 2021; Hepburn et al., 2020). Others have suggested that CP has played a too central role in policy debates to date (Rosenbloom et al., 2020). Nevertheless, CP is still considered a crucial mitigation policy which can strategically be used as part of a wider policy portfolio aimed at progressing towards sustainable, decarbonized societies (Hepburn et al., 2020; Rosenbloom et al., 2020; Van Den Bergh & Botzen, 2020). The policy influence of the literature on health impacts of CP, is likely to depend on to what extent its results can inform the real-world design of carbon pricing interventions, shaped by political economy constraints and implemented as part of a wider portfolio of measures.

Recent reviews of climate mitigation policies have synthesized evidence on social impacts (Lamb et al., 2020; Peñasco et al., 2021), without focussing on health, or on the health co-benefits of climate mitigation by sector with limited focus on specific policies (Karlsson et al., 2020; Whitmee et al., 2023).^1^

This study systematically reviews the literature on health co-benefits and trade-offs of carbon pricing, its geographical scope and the type of intervention analysed. It uses an approach based on iterative framework analysis to categorize the scenarios and outcome variables (Brunton et al., 2020). Using this approach, scenarios and outcome variables are classified based on the different types of information they provide that can be potentially relevant for policy design and implementation. Our analysis is informed by a rapid review of the literature on the political economy of carbon pricing and the role of health co-benefits in climate policy-making (see below). The evidence is synthesized narratively and, where possible, graphically, and key insights, implications and gaps are discussed. A protocol for this study was published in Open Wellcome Research (Cuevas et al., 2023).

## Methods

### Systematic literature review: search, inclusion and exclusion criteria

A search strategy was compiled in the Clarivate Analytics Web of Science Core Collection databases by an experienced information specialist (see Appendix 2). The search strategy included strings of terms and synonyms to reflect two concepts:

Concept 1: Carbon pricing. This included carbon taxes and emissions trading schemes as well as ‘indirect’ carbon pricing such as fuel taxes and subsidy removal, covering the three most important greenhouse gases (carbon dioxide, methane, nitrous oxide).
Concept 2: Health. This included general health terms as well as socioeconomic and health risk factors.

The searches were limited to those articles published in English from 2010 until October 2023. The search strategy was refined with the project team until the retrieved results reflected the scope of the project. Full details of the databases and the search strings used for each database can be found in Appendix 2. In a second step, we carried out snowballing. All citations were imported into EndNote X9 software. Duplicates were identified and removed using the method described Falconer (2018). All full texts and abstracts, as well as preliminarily included full texts were screened by two team members. Any disagreements that could not be resolved after discussion were resolved by a third team member. Inclusion and exclusion criteria are detailed in Table A1. The PRISMA diagram is in Figure A1 in the Appendix.

### Data extraction: summary mapping and iterative framework analysis

We first classified the studies according to the main type of policy intervention analysed, its geographical coverage and methods used, and the type of health outcomes reported.

Information regarding scenario design and the impacts of different policy scenarios on health co-benefits and trade-offs was extracted using a method based on qualitative framework analysis (Brunton et al., 2020). Framework analysis was developed for applied policy research but has increasingly been adapted as a method for evidence synthesis (Brunton et al., 2020; Ritchie & Spencer, 2002). Based on the steps proposed by Ritchie and Spencer (2002) the lead author first familiarized herself with the studies, focussing on scenario design, health-related outcome variables and results. Subsequently, informed by this familiarization step and by the literature reviewed in the Theoretical Background section, the relevant categories for data extraction were iteratively identified and refined (ie. optimal price levels and net benefit analysis; policy interactions; policy comparisons; policy design including revenue use; impacts on inequality)

### Theoretical background: the political economy and role of health co-benefits in the context of carbon pricing policies

In this section, we briefly review the literature on the political economy of health co-benefits and of carbon pricing, including a brief discussion of the growing literature on transformative policy approaches and its application to climate policy and health. This literature underpins a pragmatic understanding of the role of health co-benefits assessments in shaping carbon pricing policy and informs our analysis.

#### The role of health co-benefits assessments in climate policy-making

Despite the substantial health co-benefits that have been estimated from many climate mitigation interventions, several studies suggest that these estimates have had a limited impact on climate policy-making (Chang et al., 2017; Nemet et al., 2010; Remais et al., 2014; Workman et al., 2019). Nemet et al. (2010) found that monetized estimates of health co-benefits, despite being quantitatively very relevant, had enjoyed limited policy uptake. The authors also argue that the exclusion of highly uncertain climate benefits estimates from policy decision-making processes contributes to a prevailing focus on cost-minimization, as opposed to net benefit maximization.

More recent studies on the political economy of health co-benefits in climate policy have identified a wider set of barriers to impact (Workman et al., 2018, 2019). These included vested interests and the predominance of climate policy framings focussing narrowly on efficiency and market failure correction. Within these framings, the authors argue that health is considered in an ‘instrumental’ way, and its inclusion is reduced to the incorporation, via monetized and time-discounted aggregate values, into cost–benefit analysis. Workman et al. (2019) also find that, in the case of the EU, the split in responsibilities for air pollution reduction and climate change mitigation across different institutions and the limited research into the health co-benefits of mitigation interventions pose additional challenges.

#### The role of health co-benefits in the context of carbon pricing: supporting efficiency, equity, transformative change and political feasibility

Despite the relatively numerous studies quantifying the health co-benefits of CP interventions, there is a lack of evidence on the influence of health co-benefits estimates in CP policy-making processes. In the absence of this evidence, here we discuss the wider political economy of carbon pricing, considering the current logic underlying these instruments, their overall role in climate policy, the constraints faced in their implementation and the solutions proposed in the literature.

Comprehensive CP, from a theoretical perspective underpinned by neoclassical economics, has been advocated as an efficient approach to climate policy, capable of creating incentives for decarbonization throughout society at the lowest cost possible (Hepburn et al., 2020; Van Den Bergh & Botzen, 2020). Within this theoretical paradigm, policymakers simply need to identify the optimal carbon price that can correct market failures associated with? emissions (Hepburn et al., 2020) or, alternatively, the price that delivers a previously determined climate target. Monetized health co-benefits can then be incorporated into economic analysis to help produce better estimates of desirable prices and their associated net benefits (Parry et al., 2014). However, it is generally acknowledged that this idealized paradigm does not translate to real-world policy intervention (Hepburn et al., 2020; Parry et al., 2014). The existence of information asymmetries, incomplete risk markets and strong learning-by doing dynamics involved in many green solutions mean that, even within a mainstream neoclassical framing, carbon pricing is not always the most desirable intervention (Hepburn et al., 2020; Stern & Stiglitz, 2021). Beyond these considerations, there is a set of broader challenges to the notion of CP as a stand-alone efficient policy aimed at market failure correction. These can be summarized as two main related critiques: the need for transformative change and the need to prioritize political feasibility and acceptability:

The first critique, concerning the need for ‘transformative change’, (Geels & Schot, 2007) stems from a recognition of the urgency and scale of change required to meet climate targets, which involves structural changes that respond weakly if at all to marginal price changes. While some authors, in response to these considerations, have argued for a de-prioritization of CP (Rosenbloom et al., 2020), many others consider CP should still play a crucial role in climate action (Stern & Stiglitz, 2021; Van Den Bergh & Botzen, 2020). Increasingly, CP interventions are proposed and implemented as part of a coordinated policy mix aimed at delivering ‘major economic and structural transformation’ (Baranzini et al., 2017; Hepburn et al., 2020). In this approach, efficiency is one consideration among others, including equity and political feasibility, and interventions are designed to support wellbeing through both price signals and revenue use (Hepburn et al., 2020; Jenkins, 2014; Klenert et al., 2018). Although distinct in their approaches, the understanding of carbon pricing proposed by, among others, Hepburn et al. (2020), Van Den Bergh and Botzen (2020) and Klenert et al. (2018), links to the literature on ‘transformative change’ in their underlying notion that ‘fundamental change is needed’. Such a change would involve substantial societal reordering, challenging institutional structures and paradigms, in order to lead to more equitable and sustainable societies (Blythe et al., 2018; Geels & Schot, 2007; Olsson et al., 2006; Scoones et al., 2015). These proposals and the more theoretical literature on transformation share an emphasis on coordination and directionality (or policy to drive society towards a desired goal, as opposed to only correcting a market failure), and on equity, participation and engagement with a broad coalition of stakeholders as core principles (Schot & Steinmueller, 2018).

The second critique considers that political feasibility should be the priority for designing carbon pricing. An extensive literature has analysed political economy challenges for climate mitigation policies in general (Lamb et al., 2020) and carbon pricing in particular (Jenkins, 2014; Levi et al., 2020). These include the nature of climate change mitigation as a global public good,^2^ but also the perceived unfairness of carbon taxation and the fact that policy costs are concentrated on highly organized and motivated stakeholders (such as the fossil fuel or automotive industries) (Jenkins, 2014) while benefits are diffuse. Various studies (Baranzini et al., 2017; Jenkins, 2014; Klenert et al., 2018; Maestre-Andrés et al., 2019) identified the following strategies to increase policy acceptability:

- Addressing equity concerns and impacts on low-income households, through complementary interventions
- Generating visible links to local short-term benefits such as public health or energy security, where possible promoting alliances of beneficiaries (for example between health and renewables interest groups) (Workman et al., 2018).
- Making strategic and visible use of CP revenues to enhance acceptability

The rest of this article analyses whether, and how, health co-benefits assessments of carbon pricing can support political feasibility and transformative policy-making by informing not only price levels but also policy coordination, equity assessments and specific policy design, including revenue use.

## Results

Fifty eight articles were included after screening and snowballing (See PRISMA diagram, Figure A1) all of which are ex-ante modelling studies. The most common reason for article exclusion was failure to estimate or report health outcomes, or estimation of the health outcomes of non-pricing policies. Twenty six articles estimated health impacts of pricing policies of Sulfur Dioxide or NOx which are not within scope, given that sulfur dioxide cools the atmosphere and NOx does not have a clear and unambiguous climate warming effect. These articles were therefore excluded. There is an upwards trend over time of published studies, with 35 studies published in the second half of the retrieval period compared to just 20 in the first half.

### Summary mapping of the evidence

Out of the 58 included studies, *N* = 25 analysed the health impacts of carbon taxes, and *N* = 14 focussed on cap and trade policies (also referred to as Emissions Trading Schemes or ETS). A small number of studies estimated the health impacts of indirect carbon pricing including fossil fuel subsidy removal (*N* = 6) or fuel taxation (*N* = 6). Four studies reported the impact of a carbon pricing intervention, without specifying the design and a further 3 compared the health impacts of ETS versus carbon tax interventions.

All the included articles are ex-ante modelling analyses. *N* = 15 of these were computable general equilibrium models (CGE). These are ‘top-down’ models representing the entire economy, disaggregated into several sectors, and the interactions between consumers and producers across these sectors. Partial equilibrium analysis was also commonly used in several studies. Other studies employed bottom-up approaches like agent-based modelling (ABM) (Zhang & Zhang, 2020) or static-comparative cost–benefit frameworks (Parry et al., 2014).

Our analysis revealed important geographical and thematic trends, as well as areas where information is lacking.

As illustrated in Figure A2, included studies are concentrated geographically in high-income countries (and China, technically an upper-middle-income country), although there has been a growing focus on middle-income countries in recent years. Specifically, studies focus predominantly on the US (*n* = 14); China (*n* = 6); Europe or individual European countries, including the UK (*n* = 11); and other high-income countries (*n* = 3). Ten articles focus specifically on middle-income countries (Egypt, Tunisia, Mexico, India and Iran), of which 6 were published in the last two years.^3^ Fifteen studies were global or international in scope, of which one focussed on G20 and another on the top 20 emitters, which include several middle-income countries.

There is little evidence on the potential spill-overs from taxation in higher income countries on populations in lower income countries either through cross-border pollution or through impacts on trade, investment and international prices, including analysis cross-border tax adjustments are implemented.

Figure A3 maps included studies in terms of the type of intervention covered and the type of health outcomes assessed, grouped into risk factor categories. Most of the literature focuses on air pollution co-benefits, followed by dietary-related health outcomes. A small number of studies include outcomes from accidents or, in one case, from physical activity levels. The latter are driven by reinvestment of revenues into transport infrastructure.

The literature on diet-related health co-benefits forms a distinct cluster with differences in terms of methodology and scenario specification. These studies tend to focus on hypothetical demand-side taxation of specific food commodities (see Appendix Table 1 for data on sectoral coverage) as opposed to more upstream, supply-side interventions. This has been argued as a pragmatic approach adapted to the realities of the agriculture, forests and land use sector (AFOLU), where product choice is considered likely to result in large emission reductions compared to supply-side innovations, and where pricing emissions at source has been deemed too impractical and costly to implement (Springmann et al., 2017).

With two exceptions, all of the studies using CGE models have focussed on estimating air pollution outcomes. Studies focussing on dietary risk factors, on the other hand, tend to use partial equilibrium or demand models (Briggs et al., 2016; Springmann et al., 2017). Exception are Hasegawa et al. (2018), who compare a range of partial and general equilibrium models in order to estimate the impacts of economy-wide carbon taxation on food security and related health impacts, and Ortega Díaz et al. (2022) who explore the possibility of attaining health-related SDGs in Mexico using revenues from a carbon tax.

The predominant use of partial equilibrium or demand modelling in the analysis of dietary health is probably driven, to an extent, by the scenario design considerations outlined above, as well as by data limitations (notably limited data on household demand behaviour and cross-price elasticities across energy and food). Regardless of the reasons, the modelling choices made in the literature have potentially relevant impacts on the findings and insights that can be obtained from current evidence. The drivers and implications of these methodological choices will be further addressed in the discussion section.

Only one study analysed impacts on both dietary and air pollution-related outcomes, finding that dietary impacts dominate the outcomes in the study setting (Belgium) (Vandenberghe & Albrecht, 2018). However, even in this study, dietary and air pollution impacts are only analysed in relation to, respectively, food sector and energy sector pricing, and any potential cross-sectoral impacts are omitted. It is possible that the dietary impacts of comprehensive or energy sector carbon pricing initiatives are negligible. However, there is a lack of evidence on potential cross-sector health impacts of comprehensive carbon pricing.

### Co-benefit and trade-off synthesis

Here we synthesize the evidence on health co-impacts of carbon pricing, based on its policy informational content.

**Figure 2.**
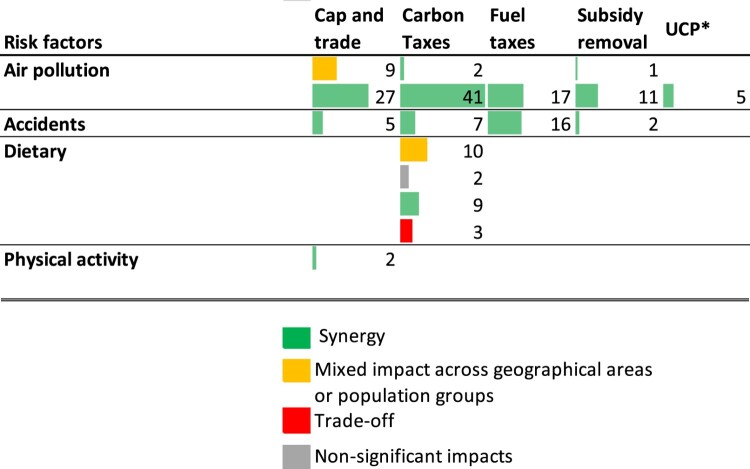
Health co-benefits and trade-offs: Summary mapping of evidence. Each observation represents a specific result. Scenarios including policy mixes or health-targeted revenue use have been removed from this figure to improve comparability and are discussed separately in this section. *UCP = unspecified carbon pricing intervention. The study models a carbon pricing intervention but does not provide further detail on its characteristics.

For every individual result (i.e. every individual scenario estimating the impact of a carbon pricing intervention), we categorize the health impact into (a) synergies only (co-benefits), (b) trade-off, mixed (e.g. synergies for some areas and trade-offs for others), and (c) no-significant impact on health. Most of the literature (Figure 2) focusses on synergies, with a small number of results pointing towards overall trade-offs from carbon taxes on diet-related health outcomes. These include mainly food insecurity-related outcomes arising from increased food prices and biofuel use but also, in one case, worsened non-communicable disease outcomes in higher income countries as a result of highly commodity-focussed carbon taxation. A slightly larger number of scenarios find both synergies and trade-offs across different countries.

#### a. Informing efficient or ‘no-regrets’ price levels and net cost estimates

We find that there is a strong emphasis in the literature on informing estimates of optimal price levels, second-best prices (Parry et al., 2015) or ‘no-regrets’ price levels (Parry et al., 2015), as well as net benefit estimates by incorporating monetized health co-benefits.

Fourteen of the 58 articles use monetized health outcomes to inform estimates of optimal, efficient, or no-regrets carbon price levels. Given that there is some inconsistency in how these terms are used in the literature, we use the terminology as follows: optimal prices as a general term to describe any price level endogenously estimated via an optimization algorithm; efficient or ‘fully efficient’ prices to refer to cases when direct climate benefits or damages are included in the optimization, together with policy costs and monetized co-benefits; No-regrets price levels are those where the costs of abatement are entirely compensated via monetized co-benefits. The most frequently used approach to monetization involves relies on Value of a Statistical Life (VSL) estimates or willingness to pay (WTP), although some studies relied on estimates of medical expenditures and productivity losses.

A larger number of studies (*N* = 29) use monetized health co-benefits to provide cost–benefit, cost-effectiveness or net cost estimates of specific price levels corresponding to existing policies or abatement targets. In most cases, monetized co-benefits are compared with abatement costs, to obtain more accurate estimates of net costs. Direct benefits from climate change mitigation are often excluded from the analysis, probably due to the uncertainty around estimates as well as the fact that they occur on very different geographical and time scales compared to costs and co-benefits (Nemet et al., 2010).

A synthesis of quantitative health co-benefits and their impacts on cost–benefit or net cost estimates is beyond the scope of this study. However, we can provide some broad qualitative insights emerging from the literature:

Firstly, most studies find that health co-benefits can offset a substantial proportion of policy costs (Coady et al., 2019), or in some cases lead to positive net benefits without accounting for climate benefits (Thompson et al., 2014; Yuan et al., 2022). As such, inclusion of health co-benefits substantially contributes to estimates of efficient or ‘no regrets’ price levels (Parry et al., 2015). For example, Vernon et al. (2021) estimate that global ‘undercharging’ for fossil fuel prices and their damages amounted to $5.9 trillion in 2020, 42% of which corresponded to health damages from air pollution. Secondly, the volume and value of co-benefits and their size relative to policy costs are highly context-specific, and tend to be larger for lower-income countries. Estimates are also highly dependent on the VSL values used to monetize outcomes as well as on modelling assumptions. Most studies rely on international VSL estimates which are adjusted based on GDP per capita. However, this approach has implications regarding equity, since health damages in higher income countries are valued more. For example, Parry et al. (2015) find that, if all countries had the same VSL, second-best CO2 prices in China would go from $63 to $207 per ton. Relying on national data as opposed to adjusted international estimates can also have large impacts on results (Li et al., 2018). Effect sizes also depend on whether models assume a more ‘long-term’ perspective, with higher price elasticities for fossil fuels or whether they adopt a short-term supply perspective, allowing only for supply responses limited by existing energy sector infrastructure and supply constraints (Luo et al., 2022; Sengupta et al., 2022; Taghvaee et al., 2022). Moreover, recent technological developments such as exponential cost reductions in photovoltaic energy storage, wind and electric vehicles were not anticipated by the studies reviewed (Way et al., 2022), which is likely to affect estimates of policy impacts in the energy and transport sectors. Comparisons across health co-benefit sizes from and different sectors and risk factor pathways in different studies are infeasible given differences in terms of approaches and assumptions. In the few studies that include co-benefits across more than one risk factor category, air pollution co-benefits are generally found to be substantially larger than those related to road traffic accidents (Parry et al., 2014).

#### b. Comparison with non-pricing mitigation policies

**Figure 3.**
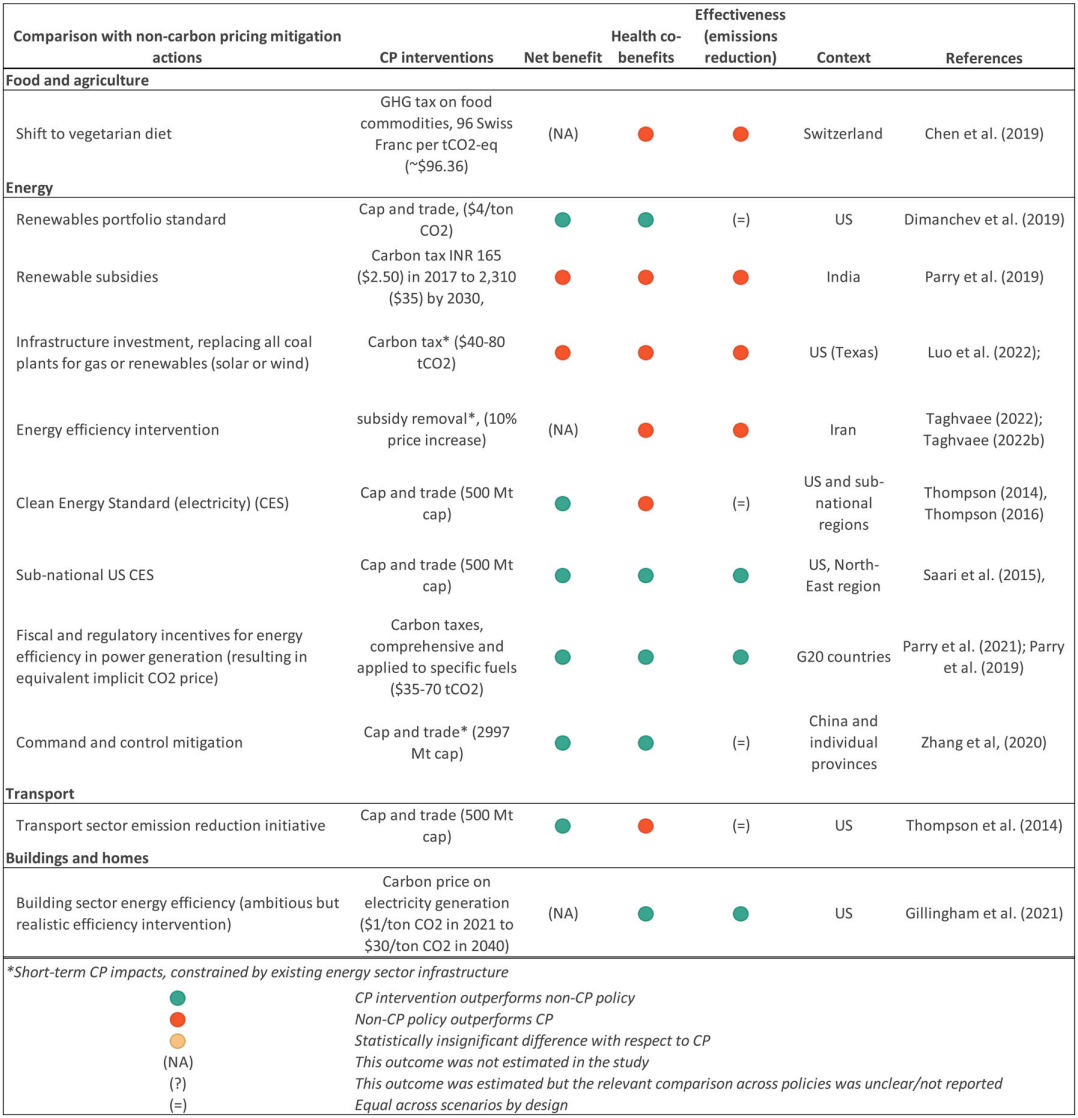
Comparison with non-pricing mitigation measures.

*N* = 11 studies explicitly compare the health co-benefits of carbon pricing with those of other mitigation policies. Figure 3 summarizes their relative performance in terms of net benefit (net impact on economic welfare measures including monetized co-benefits), health co-benefits and effectiveness in reducing emissions. Emissions trading is found to outperform a clean energy standard (CES) in the US, (Saari et al., 2014; Thompson et al., 2014, 2016) in terms of net benefit. However, there is a trade-off in terms of health co-benefits, which are generally smaller for the cap-and-trade instrument (Saari et al., 2014; Thompson et al., 2014, 2016). This is not the case across all regions, however, or for all scenarios. The CES shows larger health co-benefits than a cap-and-trade policy in a scenario where high baseline emissions are assumed, as well as for specific subnational regions (Thompson et al., 2014, 2016). A similar trade-off between efficiency and the size of health co-benefits can be found when comparing an economy-wide cap – and trade – instrument with a sectoral emissions reduction intervention targeting the transport sector (Thompson et al., 2014).

In the Chinese context, a cap-and-trade policy has been compared to a ‘command and control’ approach, finding that the former can lead to deeper emissions reductions as well as larger health co-benefits (Zhang & Zhang, 2020). However, the cap-and-trade intervention affects the geographical distribution of emissions, generating health dis-benefits in some provinces (Zhang & Zhang, 2020).

Other studies find that modelled carbon pricing interventions outperform non-pricing policies in the long run, including such as Renewables Portfolio Standards (US) (Dimanchev et al., 2019) energy efficiency interventions in the power (Parry et al., 2021) and buildings sectors (Gillingham et al., 2021). In the short run, however, air pollution gains from CP can be constrained by the existing energy infrastructure, leading to very small short-term gains compared to more structural policies such as energy efficiency interventions (Taghvaee et al., 2022; Taghvaee, Arani, et al., 2023) or replacement of coal plants by solar, gas or wind (Luo et al., 2022). Chen et al. (2019) compared carbon taxation of food commodities in Switzerland with a societal shift towards a healthier diet as recommended by national dietary guidelines, finding that the latter vastly outperforms the carbon pricing policy, both in terms GHG mitigation and health impacts. This is mainly due to the low predicted effectiveness of the GHG taxation policy in the Swiss context. Nation-wide adoption of dietary guidelines, however, is assumed and no specific policy drivers are modelled, reducing the comparability of both scenarios.

#### c. Policy design

**Figure 4.**
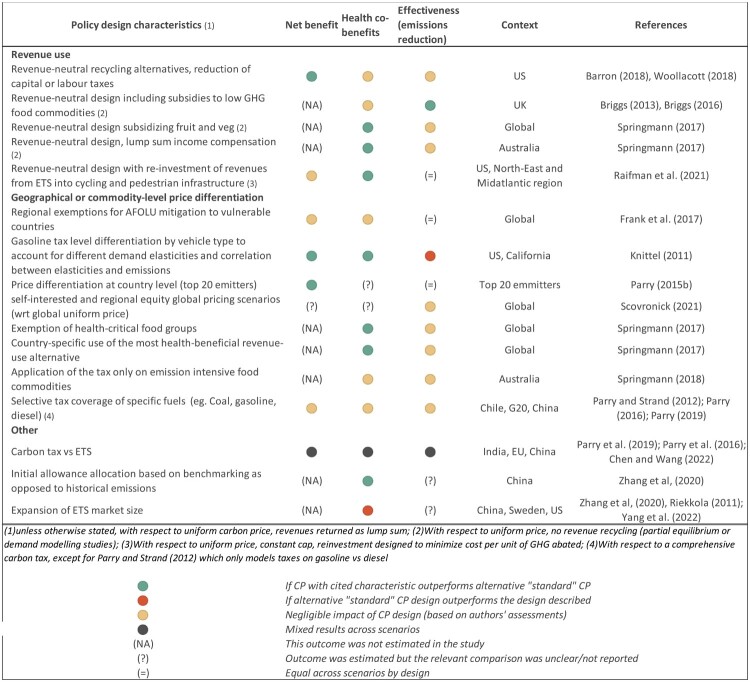
Health implications of alternative carbon pricing designs.

The literature suggests that several elements of carbon pricing intervention design beyond price levels can affect health co-benefits (Figure 4). This includes coverage (Parry et al., 2021); geographical scale (Zhang & Zhang, 2020); the implementation of uniform versus differentiated taxes either geographically or by product (Knittel & Sandle, 2011; Scovronick et al., 2021); the introduction of tax exemptions for health-related commodities (Springmann et al., 2017) and revenue utilization (Raifman et al., 2021; Springmann et al., 2017; Woollacott, 2018).

The health impacts of alternative revenue-neutral designs were explored by *n* = 6 studies. Health targeted revenue use (to invest in active transportation or subsidize healthier foods) has been found to enhance mobility (Raifman et al., 2021) and diet-related benefits while reducing food security trade-offs (Briggs et al., 2013, 2016; Springmann et al., 2017, 2018).

Non-health targeted revenue use, was found to have a negligible effect on air pollution-mediated health impacts (Barron et al., 2018; Woollacott, 2018). In the case of diet-related co-benefits, use of revenues to subsidize low-emission foods in the UK has been found to worsen non-communicable disease outcomes (Briggs et al., 2013). Springmann et al. (2017) on the other hand find that use of revenues for household income compensation slightly reduces co-benefits in high income countries but can increase health benefits in low- and middle-income regions.

More broadly, CP revenues have potential to support public healthcare and public health investments, complementing other usual funding sources within government budgets (Ortega Díaz et al., 2022).

The relative performance of carbon taxes and ETS depends on the specific design of the interventions and, in particular, on relative coverage and price pathways over time (Chen & Wang, 2023; Parry et al., 2016, 2019). A comprehensive carbon tax would in principle have larger health benefits and be more efficient than a similarly stringent intervention modelled on existing emissions trading schemes which, due to practical constraints, tend to cover only the power sector and large industry emitters (Parry et al., 2016, 2019).

Tax differentiation has also been explored by several studies (*N* = 8). Tax differentiation across commodities has been shown to improve health co-benefits at a small trade-off in terms of mitigation effectiveness (Springmann et al., 2017, 2018). Knittel and Sandle (2011) show that optimal heterogeneous carbon taxation in the transport sector to account for the correlation between price elasticity and emissions of different types of vehicles improves health co-benefits and net benefit. However, they also find that it leads to smaller emissions reductions overall, when compared to second-best homogeneous taxation. Geographic tax exemptions or differentiation for vulnerable regions have been explored as well, in relation to air pollution and diet-related impacts, with mixed findings. Frank et al. (2019) find that, for a given total emission reduction target, regional exemptions might lead to inefficient mitigation in other regions, leading to costs that are transmitted through international trade thus increasing food insecurity. Scovronick et al. (2021) find that international price differentiation in nationally self-interested scenarios and cooperative scenarios that prioritize regional equity leads to complex and region-specific impacts on climate and health co-benefits. Overall, inclusion of health co-benefits increases mitigation in all scenarios, although is not generally sufficient on its own to prevent excessive temperature increases in a purely self-interested mitigation scenario, where countries price CO2 based on local air pollution damages.

Zhang and Zhang (2020) find that expansion of market scale in China from provincial to regional to national can result in reduced air quality co-benefits, with specific provinces that become net purchasers of emission permits subsequently experiencing reduced air quality. Initial allocation of permits also has an impact, with allocation based on benchmarking resulting in higher co-benefits when compared to allocation based on historical emissions.

#### d. Policy interactions

**Figure 5.**
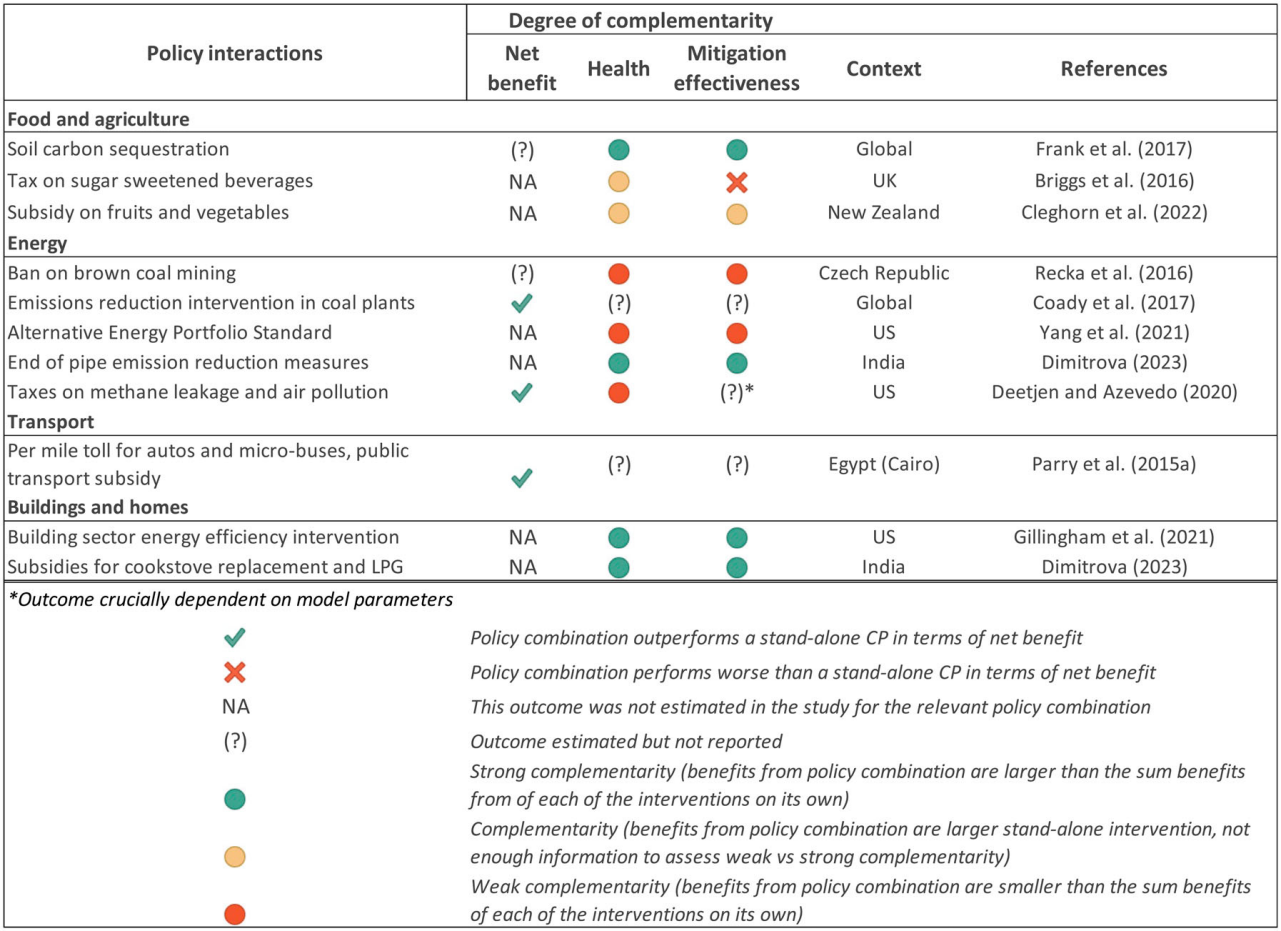
Health, efficiency and effectiveness implications of policy interactions.

*N* = 10 studies have analysed how health co-benefits are affected by the interaction with other policies (Figure 5). The findings are highly study and scenario-specific but highlight how additional decarbonization actions can reduce expected co-benefits attributable to each intervention alone (Recka & Scasny, 2016; Yang et al., 2021) while complementary policies can enhance co-benefits by directing demand substitution away from harmful products.^4^ This can include sugar taxes to minimize replacement by unhealthy foods in response to taxes on high GHG emitting foods (Briggs et al., 2016), or subsidies on clean cookstoves or LPG in India to avoid use of comparatively ‘dirtier’ cooking fuels which would worsen indoor air pollution (Dimitrova et al., 2022). In the food and agriculture sector, combination with soil carbon sequestration have been found to mitigate food security trade-offs (Frank et al., 2017), by keeping a larger proportion of land under production in CP scenarios. CP can also exhibit strong complementarity with building sector efficiency interventions, simply by improving outdoor air quality and thus mitigating the negative impacts of the latter intervention on indoor air pollution (Gillingham et al., 2021). In the transport sector, Parry and Timilsina (2015) find that, when health co-benefits are considered, the most efficient policy combines gasoline taxation with per km vehicle tolls and subsidization of public transport. In the power generation sector, combining CO2 taxes with taxes on methane leakage and air pollution can also improve policy efficiency and health co-benefits at a small or no trade-off in terms of mitigation effectiveness (Deetjen & Azevedo, 2020). Overall, the findings in this section illustrate how analysis of policy interactions could potentially support coordinated policy interventions, helping design policy mixes that leverage the potential for co-benefits while addressing trade-offs and protecting the most vulnerable populations.

#### e. Health inequalities and trade-offs

*N* = 20 studies have analysed differences in outcomes and costs across geographical regions. We find that the co-benefits and costs of carbon pricing can be very unevenly distributed across geographical areas. In many cases, even the regions with least favourable cost–benefit ratios experience positive net benefits and geographical differences merely suggest the need to implement context-specific approaches and geographically differentiated prices (Barron et al., 2018; Klaiber et al., 2023; Saari et al., 2014). Co-benefits from air pollution reductions tend to be larger in LMICs and in certain regions (China, Russia, Central and Eastern Europe, South, South East and East Asia) as the baseline air quality tends to be worse and there is a higher reliance on burning coal as fuel (Klaiber et al., 2023; Lelieveld et al., 2023; Markandya et al., 2018; Parry et al., 2015, 2021). This also applies at a sub-national level, with co-benefits concentrating on coal-reliant regions in the US (Barron et al., 2018; Saari et al., 2014; Thompson et al., 2016), and in India (Dimitrova et al., 2022).

There is some evidence, moreover, that in the absence of adequate compensatory measures, CP can lead to localized health damages related to worsened exposure to air pollution. This can occur through leakages of co-pollutants, which happen as production and sales move to areas not covered by the intervention (Thompson et al., 2016; Yang et al., 2021). Other mechanisms include increased concentration of emissions in areas which, although covered by the intervention, have lower marginal costs per ton of CO2 emitted (Zhang & Zhang, 2020) or through substitution by dirty fuels in lower income areas (Klaiber et al., 2023).

A small number of recent articles have analysed how carbon pricing affects health disparities driven by unequal exposure to outdoor air pollutants across socioeconomic status, income, ethnicity and gender. Both Picciano et al. (2022) and Luo et al. (2022) find that, while producing considerable co-benefits across all socioeconomic groups, CP does not reduce health disparities, suggesting the need for additional targeted interventions to address equity. Several studies have found that CP implemented in LMIC settings without any compensatory interventions could potentially lead to negative health impacts, increasing food insecurity (Frank et al., 2017; Hasegawa et al., 2018; Springmann et al., 2017) or aggravating household air pollution in disadvantaged households in India, resulting in increased rates of stunting, especially for girls (Dimitrova et al., 2022).

A more complete understanding of health trade-offs is indispensable for the adequate design of compensatory and redistributive policies which include targeted subsidies or income transfers, and complementary local emissions regulations.

## Discussion

### Health co-benefits and trade-offs: scenarios, impact estimates and policy

Our analysis highlights the predominant focus on optimization of carbon price levels and cost–benefit, cost-effectiveness or net benefit estimation for specific abatement targets and their corresponding carbon prices. This is perhaps unsurprising, given that CP was initially advocated by economists largely on the premise of its theoretical efficiency compared to other interventions, and the estimation of efficient pricing for market failure correction has been considered a central component of CP design (Hepburn et al., 2020). Most studies reviewed find that the inclusion of monetized health effects affects estimates of efficient, second best or ‘no regrets’ carbon pricing levels. Moreover, several studies show that the levels of ‘no regrets’ or ‘own interest’ carbon pricing are substantial in many countries, notably including LMICs (Parry et al., 2015). Therefore, this information can, in theory, have substantial policy implications. Specific values for optimal prices and net benefits, however, depend to a large extent on modelling assumptions and, in particular, on VSL estimates, limiting the potential for policy uptake. The political economy literature has also highlighted a number of factors that limit the degree to which these estimates incentivise adoption of higher carbon price levels or their implementation in new contexts, including the impact of policy costs on a small number of highly organized industry stakeholders that can oppose change, while benefits are highly diffuse. A small number of studies provide metrics that reflect actual budgetary implications for governments and health departments (Chen & Wang, 2023; Cleghorn et al., 2022) who, as highly organized and motivated stakeholders could constitute powerful advocates for ambitious carbon price levels (Jenkins, 2014; Nemet et al., 2010; Workman et al., 2018).

In addition to informing price levels, the evidence reviewed suggests that there is substantial scope to boost health co-benefits and mitigate trade-offs through careful policy design including: health-targeted revenue-neutral designs; combination with policies such as those supporting income compensation, food security and access to clean affordable fuels for vulnerable households; taxation or subsidy policies that encourage healthy demand substitution in response to price changes; air pollution regulatory strengthening to complement cap and trade. There are also potential trade-offs in terms of policy design e.g. exemption of specific commodities or health-promoting revenue investment might improve health co-benefits at the expense of some efficiency or effectiveness in the policy’s primary goal of emission abatement. Additionally, some results suggest that there is some potential for counter-intuitive impacts if health or equity-based exemptions of certain regions, sectors or commodities lead to efficiency losses in international food systems which might exacerbate food insecurity, even in those regions the exemptions sought to protect (Frank et al., 2017).

Many of these strategies have the potential to address, at least partially, some of the main political feasibility constraints facing CP adoption as identified in the political economy literature. In particular, health-targeted revenue uses including subsidies for public transport, clean cookstoves or healthy foods, while not commonly discussed in the wider literature on CP, can achieve wide social acceptability and visibility (Klenert et al., 2018). At the same time, they can generate organized constituencies of beneficiaries (Jenkins, 2014) and address equity concerns (Maestre-Andrés et al., 2019). Another opportunity lies in the implementation of synergistic policy mixes, with CP included as part of a ‘transformative’ climate agenda (Hepburn et al., 2020), emphasizing coordination and policy directionality (Geels & Schot, 2007) as opposed to just market failure correction. This approach can be hampered by the division of responsibilities across health departments and those with responsibilities over climate (Workman et al., 2019).- However, the identification of policy combinations that exhibit strong complementarities both in terms of climate mitigation and health co-benefits (e.g. regulations addressing localized air pollution damages or sugar taxation to avoid unhealthy substitutions in response to price changes) might provide incentives for increased coordination across government departments.

Overall, there is a need for further evidence in each of these areas. Taken together, however, this range of information highlights the importance of considering context, specific policy design, policy interactions, health inequalities and trade-offs from carbon pricing. As such, it can feed into the design of coordinated policymaking, supporting policy prioritization, health-sensitive design, deliberate choice of policy combinations that can boost health co-benefits and the inclusion of mechanisms that reduce health inequalities.

Climate mitigation in general and carbon pricing in particular are highly complex and contested areas of policymaking. A wide range of societal concerns, as well as, of course, vested interests, influence policy outcomes, and health co-benefits might only be one among others. While the maximization of health co-benefits is not the primary goal of climate policy, it can enter policy decisions in various ways, via formal assessments and otherwise, including as a co-benefit or as a constraint.

### Gaps in the literature and opportunities for further research

This study highlights important gaps in the evidence around carbon pricing and health, as well as relevant clusters of evidence. The predominant focus on high-income countries (and China) and relative lack of evidence focussing on middle-income countries constitutes an important gap. Although higher-income countries are amongst the largest contributors to global emissions, the relative neglect of middle-income countries represents an important gap in the literature for various reasons: Firstly, recent data show that a growing number of countries in Asia and Latin-America have implemented or scheduled carbon pricing policies (World Bank, 2022). Secondly, some studies that include countries across income categories, as well as a small number of recent single-country studies (Dimitrova et al., 2022; Ortega Díaz et al., 2022; Taghvaee, Nodehi, et al., 2023), find comparatively large health co-benefits for middle-income countries, which often stand to gain more from air pollution reduction (Parry et al., 2015), but which can also experience trade-offs, particularly in relation to food insecurity, undernutrition (Springmann et al., 2017) or household air pollution (Dimitrova et al., 2022). This type of information can have multiple potential policy implications, from the levels of carbon pricing in countries’ own interest to the importance of implementing safety nets or context-specific designs to avoid trade-offs. Given the context-specificity of health co-benefits, detailed single-country studies are necessary in addition to global multi-country studies which often rely on generalizations.

In terms of the risk factors and outcomes analysed, there are several potential explanations for our finding that the bulk of the evidence focusses on ambient (outdoor) air pollution and dietary risk factors. Firstly, outdoor air pollution and dietary impacts are likely to be among the largest health co-benefits, based on the limited comparisons with other potential co-benefits available in the literature (Coady et al., 2017; Parry & Timilsina, 2015). However, recent studies have suggested that the physical activity benefits of climate mitigation could be very large (Whitmee et al., 2023), and perhaps even larger than air pollution co-benefits in some settings (Hamilton et al., 2021). The size of air pollution co-benefits, however, can greatly depend on the exposure-response relationships assumed, which could also affect the relative size of different types of co-benefits (Pozzer et al., 2023). It is also possible that air pollution exposure and diets are most frequently assessed because they are the most direct health impacts from pricing interventions, for which pathways are widely understood and where it is possible to design ex-ante models based on relatively straightforward assumptions. Mobility and energy poverty are potentially relevant pathways of impact which have been discussed in the literature (Parry, 2014). Mobility behaviour, however, is highly complex and contextual, and behavioural responses to price increases are likely to depend crucially on existing transport infrastructure. Evidence suggests, moreover, that mere price changes are unlikely to lead to mass switches across transport modes (OECD, 2022).

Other outcomes such as health impacts from energy insecurity or household (indoor) air pollution for vulnerable households deserve further investigation, particularly in the current context of energy crisis, and despite the potential methodological difficulties and inherent heterogeneities (Parry, 2014).

In addition to gaps concerning specific risk factors there is a lack of research on cross-sectoral impacts (e.g. of carbon pricing outside the AFOLU sector on diet-related health outcomes). Likewise, there is a lack of studies including a comprehensive range of health co-benefits and trade-offs, elicited systematically either through preliminary literature review, stakeholder involvement or other methods. In the broader context of mitigation co-benefits, this is likely to limit the policy impact of research (Remais et al., 2014).

There is a need for more studies focussing on middle-income countries and addressing a wider range of health risk factors, as well as more ex-post evidence of carbon pricing interventions and their health impacts. There is a literature on ex-post associations between fossil fuel prices and emissions, and between prices and heath. However, these studies do not simultaneously include both outcomes. The inclusion restriction used in this review that studies should include both GHG and health outcomes is useful, allowing us discuss relationships between effectiveness, efficiency and health co-benefits and to frame fossil fuel price changes in the context of climate change mitigation. However, it implies the exclusion of some useful literature, including ex-post studies which could be addressed in a separate review.

## Conclusion

This literature review provides a comprehensive synthesis of the health co-benefits of carbon pricing. Informed by the literature on the political economy of carbon pricing, findings are discussed in terms of their relevance for real-world policy-making.

In particular, the net benefits and optimal carbon price levels resulting from internalizing health co-benefits estimates, can be useful in advocating for higher carbon prices. This is particularly the case in middle-income countries and coal-reliant regions in high income countries, where air pollution co-benefits tend to be larger. In the very short term, and in energy systems with strong inefficiencies and constraints, other policies targeting energy system structural upgrades such as by supporting electrification are likely to yield larger health co-benefits, however, and CP should be considered as a complementary policy. Uncertainties in estimating health co-benefits are acknowledged but should not be a barrier to their use to inform CP policies, particularly when these policies include mechanisms to minimize trade-offs, including potential equity implications.

Earmarking part of the revenues from CP for health interventions can also be considered as an option to boost public acceptability and political support, encouraging support for CP from highly organized health-sector stakeholders and potentially reduce inequities. In particular, use of funds to support vulnerable households and avoid trade-offs can boost net co-benefits while increasing equity and political feasibility. This can include transfers to support nutrition and energy security, public transport and affordability of clean cooking alternatives.

Beyond revenue use, policy makers can promote health co-benefits in others ways, including through complementary taxation to encourage healthy substitution patterns in response to carbon prices, or (partial) exemption for vulnerable regions or commodities that are key for health. Complementary air pollution regulations can also be necessary to avoid localized health damages from CP as a result of carbon leakage or concentration of emissions in areas with lower marginal costs. While some of these policies can be strongly synergistic, in other cases there can be trade-offs with policy effectiveness and efficiency which should be considered on a case by case basis.

Equity is a central concern for the successful adoption of CP. When it comes to health, ethnic and gender dimensions are crucial and intersect with socioeconomic factors. CP implementation, even when delivering co-benefits for all groups, generally will not reduce health inequalities. Broader strategies beyond CP and indeed beyond climate mitigation are necessary to tackle health inequalities in air pollution exposure, diets and other risk factors.

Overall, existing evidence supports the importance of embedding context-specific consideration of health co-benefits and trade-offs in the design and implementation of carbon pricing as part of broader climate policy mixes, supported by increased cooperation across government authorities with responsibilities for health and climate. This type of approach can support a move towards health- sensitive carbon pricing design, improving acceptability and delivering both health and climate benefits.

## Supplementary Material

Supplemental Material

## Notes

1 The report by Whitmee et al. (2023) includes a short section on carbon pricing which is based on the analysis of a sub-set of the studies included in the present article, carried out by the same authors.

2 Global public goods are non-rival and non-excludable, meaning that everyone benefits from the mitigation efforts of each country, so individual countries in principle have incentives to ‘free-ride’, benefiting from others´ emission reduction efforts while continuing to emit themselves (Bättig & Bernauer, 2009).

3 However, we note that this includes 3 studies on Iran from the same authors which are similar in terms of approach and findings. Nevertheless, this is an improvement compared to only 4 single- country studies on LMIC in the previous decade.

4 We refer to weak and strong complementarity to differentiate between cases where impacts are additive and cases where joint implementation leads to benefits that are larger than the sum of the impacts of individually implemented policies. Others use slightly different terminology, referring instead to ‘complementarity and synergy’ (Howlett & Rayner, 2007) or ‘net complementarity’ (Dimitrova et al., 2022).
