# Supplementary material for: Health co-benefits and trade-offs of carbon pricing: a narrative synthesis
Source: Clim Policy. 2024 Jun 2;24(10):1346–64. doi: 10.1080/14693062.2024.2356822 (PMC11523918; doi:10.1080/14693062.2024.2356822)
Supplement: Supplemental Material [file TCPO_A_2356822_SM1264.docx]

# **Health co-benefits and trade-offs of carbon pricing: A narrative synthesis**

Soledad Cuevas ₁,_4_, Daniel Nachtigall ₂, Aimee Aguilar Jaber ₂, Kristine Belesova _1_,_3_ Jane Falconer ₁, Andy Haines ₁,Tamzin Reynolds ₁, Tobias Magnus Schuster_1_, Sarah Whitmee_1_, Rosemary Green ₁
1 London School of Hygiene and Tropical Medicine
2 OECD

3 Imperial College London

4 Instituto de Economía, Geografía y Demografía (IEGD), Consejo Superior de Investigaciones Científicas (CSIC)

# Appendix 1. Supplementary figures and tables

**Table A1 Inclusion and exclusion criteria**

|  | Included | Excluded |
| --- | --- | --- |
| Population | Studies analysing impacts at a local, sub-national, national or international level | None |
| Intervention | Direct and indirect carbon pricing interventions: Carbon taxes, emissions trading schemes, carbon crediting. Indirect: Taxes on fuels, fossil fuel subsidy removal | Other climate change mitigation policy, where separate results are not provided for carbon pricing |
| Comparator | Affected versus unaffected geographical areas, before and after, intervention scenario versus counterfactual without carbon pricing intervention | Descriptive studies (eg. identifying potentially relevant outcomes) |
| Outcomes | GHG emissions (main gases, CO2, CH4 and N2O) AND  Impacts on health outcomes. These would include  Any morbidity or mortality outcome, including burden of disease, QUALYs, DALYs, monetized estimates of health outcomes | Does not include impacts on GHG emissions  OR does not include health outcomes |
| Study design | Ex-post quantitative studies, ex-ante applied modelling studies. Empirical qualitative studies. Relevant systematic reviews were checked to identify potential studies for inclusion. | Theoretical studies |
| Year of publication | 2010 or after | Before 2010 |
| Language | English language | Other |

**Figure A1 Simplified Prisma diagram**


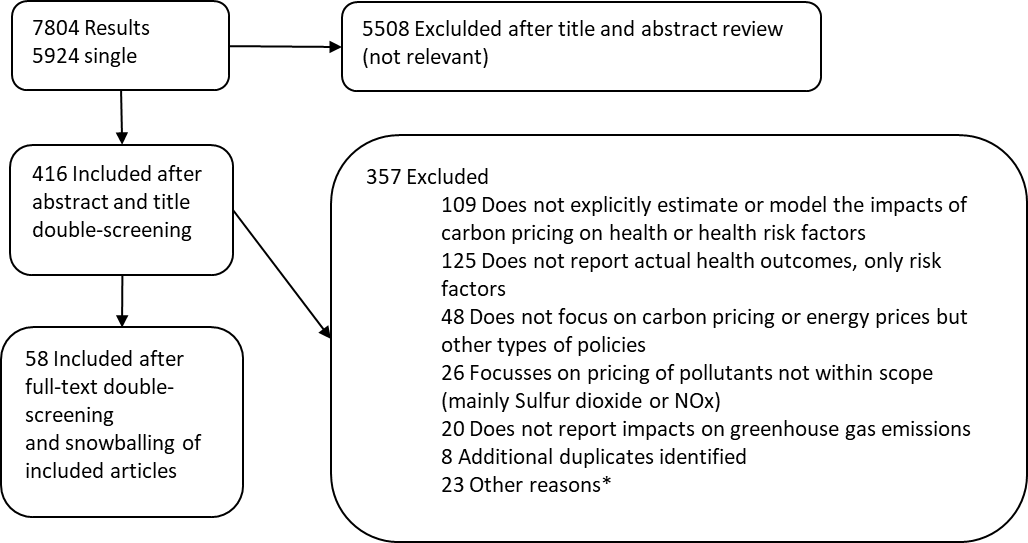


**Figure A2 Geographical coverage of included studies**

**Figure A3 Summary map of evidence – Types of intervention and risk factor categories**

**
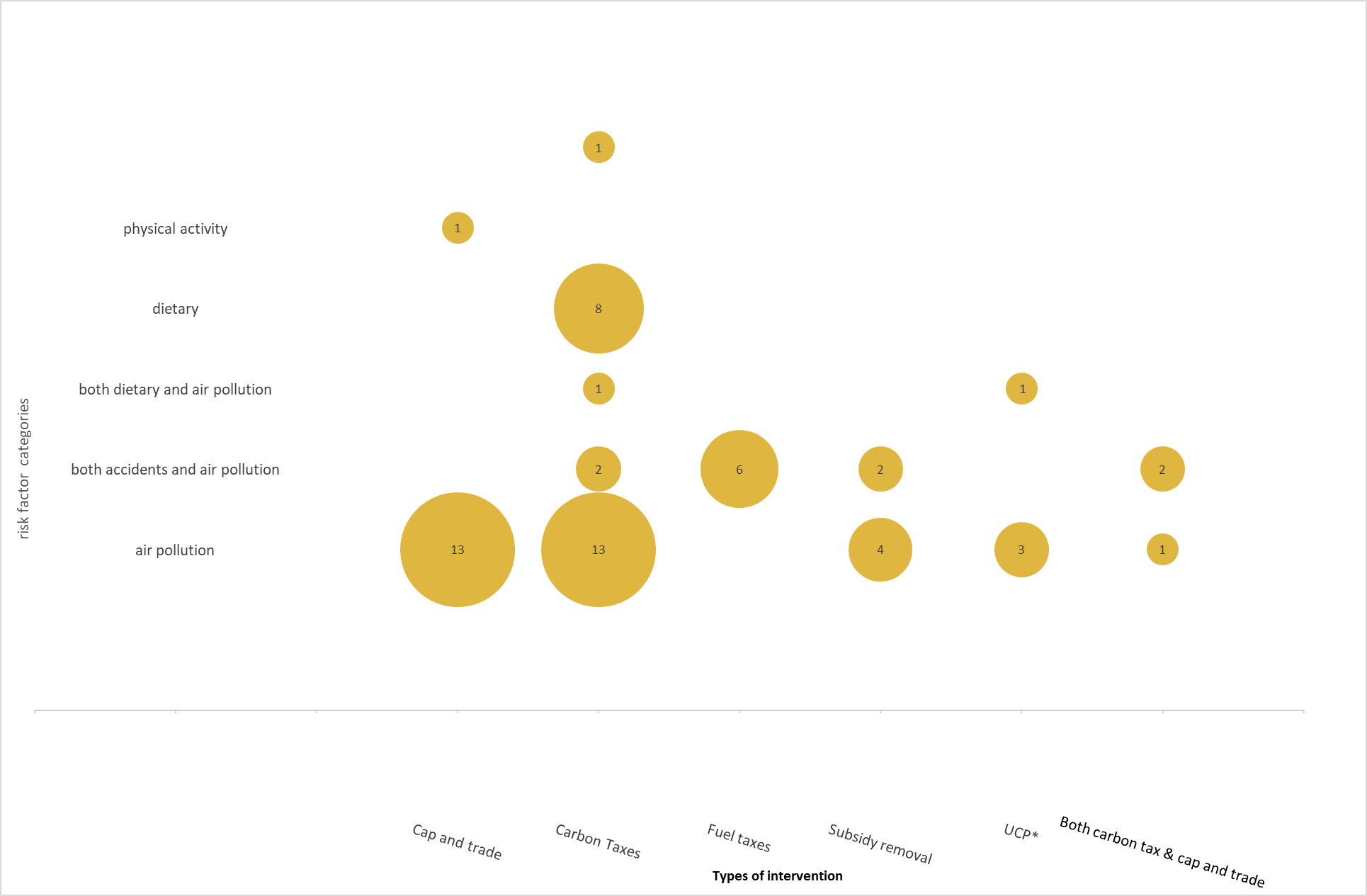
**

Each observation represents one study. *UCP = Unspecified carbon pricing intervention. The study models a carbon pricing intervention but does not provide further detail on its characteristics

**Appendix table A2. Included studies**

| Study reference | Country/Region | Intervention | Sectoral coverage | Risk factor category | Outcome Type |
| --- | --- | --- | --- | --- | --- |
| Barron et al. 2018 | US | Carbon Taxes | Comprehensive or Economy Wide | Air pollution | Both monetized and non-monetized (actual) health outcomes |
| Briggs et al. 2013 | UK | Carbon Taxes | Food & or Agriculture | Dietary | Only actual health outcomes (not monetized) |
| Briggs et al. 2016 | UK | Carbon Taxes | Food & or Agriculture | Dietary | Only actual health outcomes (not monetized) |
| Chang et al. 2020 | China | Cap and trade | Comprehensive or Economy Wide | Air pollution | Both monetized and non-monetized (actual) health outcomes |
| Chemingui and Thabet 2014 | Tunisia | Carbon Taxes | Comprehensive or Economy Wide | Air pollution | Monetized health outcomes only |
| Chen et al. 2019 | Switzerland | Carbon Taxes | Food & or Agriculture | Dietary | Only actual health outcomes (not monetized) |
| Coady et al. 2017 | Global (155 countries) | Subsidy removal | Energy & Industry | Both accidents and air pollution | Both monetized and non-monetized (actual) health outcomes |
| Coady et al. 2019 | International/Global | Subsidy removal | Energy & Industry | Both accidents and air pollution | Both monetized and non-monetized (actual) health outcomes |
| Deetjen and Azevedo 2020 | US | Carbon Taxes | Energy | Air pollution | Monetized health outcomes only |
| Dimanchevi et al. 2019 | US | Cap and trade | Comprehensive or Economy Wide | Air pollution | Both monetized and non-monetized (actual) health outcomes |
| Frank et. al 2017 | Global | Carbon Taxes | Food & or Agriculture | Dietary | Only actual health outcomes (not monetized) |
| Hasegawa et al. 2018 | Global | Carbon Taxes | Food & or Agriculture | Dietary | Only actual health outcomes (not monetized) |
| Knittel et al. 2011 | California, US | Carbon Taxes | Transport | Air pollution | Monetized health outcomes only |
| Korkmaz et al. 2020 | EU/Europe | Carbon price-unspecified | Energy & Industry | Air pollution | Monetized health outcomes only |
| Li et al. 2018 | China | Carbon price-unspecified | Energy & Industry | Air pollution | Both monetized and non-monetized (actual) health outcomes |
| Markandya et al. 2018 | International/Global | Carbon Taxes | Comprehensive or Economy Wide | Air pollution | Both monetized and non-monetized (actual) health outcomes |
| Parry et al. 2014 | International/Global | Carbon Taxes | Coal Gas motor Fuels | Both accidents and air pollution | Both monetized and non-monetized (actual) health outcomes |
| Parry and Timilsina. 2015a | Greater Cairo, Egypt | Fuel taxes | Transport | Both accidents and air pollution | Monetized only (actual health outcomes in appendix) |
| Parry et al. 2015b | Top 20 emmitters | Fuel taxes | Comprehensive or Economy Wide | Both accidents and air pollution | Monetized health outcomes only |
| Parry et al. 2016 | China | Carbon Taxes | Power & transport | Both accidents and air pollution | Both monetized and non-monetized (actual) health outcomes |
| Parry et al. 2021 | G20 countries | Fuel taxes | Comprehensive or Economy Wide | Both accidents and air pollution | Both monetized and non-monetized (actual) health outcomes |
| Parry et al. 2016b | EU/Europe | Fuel taxes | Transport and heating | Both accidents and air pollution | Both monetized and non-monetized (actual) health outcomes |
| Raifman et al. 2021 | US, Northeast and Mid-Atlantic region | Cap and trade | Transport | Physical activity | Both monetized and non-monetized (actual) health outcomes |
| Recka et al. 2016 | Czech Republic | Cap and trade | Energy & IndustryPower manufacturing & airlines | Air pollution | Monetized health outcomes only |
| Saari et al. 2015 | US and regions | Cap and trade | Comprehensive or Economy Wide | Air pollution | Monetized only (actual health outcomes in appendix) |
| Scasny the 2015 | Europe | Carbon Taxes | Comprehensive or Economy Wide | Air pollution | Monetized health outcomes only |
| Scovronick et al. 2021 | International/Global | Cap and trade | Comprehensive or Economy Wide | Air pollution | Monetized health outcomes only |
| Springmann Mos. 2017 | International/Global | Carbon Taxes | Food & or Agriculture | Dietary | Only actual health outcomes (not monetized) |
| Springmann et al. 2018 | Australia | Carbon Taxes | Food & or Agriculture | Dietary | Both monetized and non-monetized (actual) health outcomes |
| Thompson et al. 2016 | US | Cap and trade | Comprehensive or Economy Wide | Air pollution | Both monetized and non-monetized (actual) health outcomesMonetized health outcomes only |
| Thompson et al. 2014 | US, Northeast and other regions (17 states in total, see p. 992 for the list) | Cap and trade | Comprehensive or Economy Wide | Air pollution | Both monetized and non-monetized (actual) health outcomes |
| Vandenberghe et al. 2018 | Belgium | Carbon Taxes | Energy & food | Both dietary and air pollution | Both monetized and non-monetized (actual) health outcomes |
| Woollacott et al. 2018 | United States | Carbon Taxes | Comprehensive or Economy Wide | Air pollution | Both monetized and non-monetized (actual) health outcomes |
| Wyrwa et al. 2015 | Poland | Cap and trade | Energy & IndustryPower manufacturing & airlines | Air pollution | Only actual health outcomes (not monetized) |
| Yang et al. 2018 | China | Carbon Taxes | Energy & Industry | Air pollution | Monetized health outcomes only |
| Zhang et al. 2020 | China | Cap and trade | Energy & Industry | Air pollution | Both monetized and non-monetized (actual) health outcomes |
| Garcia Menendez 2015 | International/Global | Carbon Taxes | Comprehensive or Economy Wide | Air pollution | Both monetized and non-monetized (actual) health outcomes |
| Parry and Strand 2012 | Chile | Fuel taxes | Transport | Both accidents and air pollution | Monetized health outcomes only |
| Parry and Timilsina 2010 | Mexico City | Fuel taxes | Transport | Both accidents and air pollution | Monetized health outcomes only |
| Parry et al. 2019 | India | Both ETS and carbon tax | Energy and transport | Both accidents and air pollution | Both monetized and non-monetized (actual) health outcomes |
| Riekkola et al. 2011 | Sweden | Cap and trade | Comprehensive or Economy Wide | Air pollution | Monetized health outcomes only |
| West et al. 2013 | International/Global | Carbon price-unspecified | Comprehensive or Economy Wide | Air pollution | Both monetized and non-monetized (actual) health outcomes |
| Chen and Wang 2022 | China | Both cap and trade and carbon tax | Comprehensive or Economy Wide | Air pollution | Monetized health outcomes only |
| Cleghorn et al. 2022 | New Zealand | Carbon taxes | Food & or Agriculture | Dietary | Both monetized and not monetized |
| Gillingham et al. 2021 | USA | Carbon price-unspecified | Building sector | Air pollution | Only actual health outcomes (not monetized) |
| Luo et al. 2022 | USA (Texas) | Carbon Taxes | Energy | Air pollution | Both monetized and non monetized |
| Sengupta et al. 2022 | India | Carbon Taxes | Energy | Air pollution | Only actual health outcomes (not monetized) |
| Yang et al. 2021 | USA (Pennsylvania and Northeast) | Carbon taxes | Energy | Air pollution | Monetized health outcomes only |
| Vernon et al. 2021 | International/Global | Fuel taxes | Coal, natural gas, road diesel, gasoline | Both accidents and air pollution | Monetized health outcomes only |
| Picciano et al. 2023 | USA | Cap and trade | Energy | Air pollution | Only actual health outcomes |
| Taghavee et al. 2022 | Iran | Subsidy removal | Diesel | Air pollution | Only actual health outcomes |
| Taghavee et al. 2022 | Iran | Subsidy removal | Diesel, gasoline, fuel oil, Liquefied Petroleum Gas (LPG), and kerosene | Air pollution | Only actual health outcomes |
| Taghavee et al. 2023 | Iran | Subsidy removal | Diesel, gasoline, fuel oil, Liquefied Petroleum Gas (LPG), and kerosene | Air pollution | Only actual health outcomes |
| Soergel et al 2023 | International/Global | Carbon price-unspecified | Energy | Both dietary and air pollution | Only actual health outcomes |
| Ortega-Diaz et al. 2023 | Mexico | Carbon taxes | Comprehensive | Expenditure on public services and infrastructure for education, healthcare, sewage and potable water | Only actual health outcomes |
| Klaiber et al. 2023 | International/Global | Subsidy removal | Energy | Air pollution | Only actual health outcomes |
| Yuan et al. 2023 | USA | Cap and trade | Comprehensive | Air pollution | Monetized health outcomes only |
| Dimitrova et al. 2023 | India | Carbon taxes | Comprehensive | Air pollution | Only actual health outcomes |

# **Appendix 2.** Final search strategies

Carbon pricing and health: Pathways of impact and opportunities for transformative change
Updated search strategies, October 2023

Table of Contents

[1 Search methodology 2](file:///C:\Users\usuario\Downloads\2023-10%20Carbon%20pricing%20review%20updated%20search%20strategies%20(1).docx#_Toc149205780)

[2 Databases 2](file:///C:\Users\usuario\Downloads\2023-10%20Carbon%20pricing%20review%20updated%20search%20strategies%20(1).docx#_Toc149205781)

[2.1 Information management and deduplication 3](file:///C:\Users\usuario\Downloads\2023-10%20Carbon%20pricing%20review%20updated%20search%20strategies%20(1).docx#_Toc149205782)

[3 Results 4](file:///C:\Users\usuario\Downloads\2023-10%20Carbon%20pricing%20review%20updated%20search%20strategies%20(1).docx#_Toc149205783)

[4 References 5](file:///C:\Users\usuario\Downloads\2023-10%20Carbon%20pricing%20review%20updated%20search%20strategies%20(1).docx#_Toc149205784)

[5 Appendix: Search strategies 5](file:///C:\Users\usuario\Downloads\2023-10%20Carbon%20pricing%20review%20updated%20search%20strategies%20(1).docx#_Toc149205785)

[5.1 Medline ALL 5](file:///C:\Users\usuario\Downloads\2023-10%20Carbon%20pricing%20review%20updated%20search%20strategies%20(1).docx#_Toc149205786)

[5.2 Embase 8](file:///C:\Users\usuario\Downloads\2023-10%20Carbon%20pricing%20review%20updated%20search%20strategies%20(1).docx#_Toc149205787)

[5.3 Global Health 11](file:///C:\Users\usuario\Downloads\2023-10%20Carbon%20pricing%20review%20updated%20search%20strategies%20(1).docx#_Toc149205788)

[5.4 Econlit 13](file:///C:\Users\usuario\Downloads\2023-10%20Carbon%20pricing%20review%20updated%20search%20strategies%20(1).docx#_Toc149205789)

[5.5 Africa-Wide Information 15](file:///C:\Users\usuario\Downloads\2023-10%20Carbon%20pricing%20review%20updated%20search%20strategies%20(1).docx#_Toc149205790)

[5.6 GreenFILE 16](file:///C:\Users\usuario\Downloads\2023-10%20Carbon%20pricing%20review%20updated%20search%20strategies%20(1).docx#_Toc149205791)

[5.7 Web of Science Core Collection 18](file:///C:\Users\usuario\Downloads\2023-10%20Carbon%20pricing%20review%20updated%20search%20strategies%20(1).docx#_Toc149205792)

[5.8 Korean Journal Database 21](file:///C:\Users\usuario\Downloads\2023-10%20Carbon%20pricing%20review%20updated%20search%20strategies%20(1).docx#_Toc149205793)

[5.9 SciELO Citation Index 23](file:///C:\Users\usuario\Downloads\2023-10%20Carbon%20pricing%20review%20updated%20search%20strategies%20(1).docx#_Toc149205794)

# Search methodology

A draft search strategy was compiled in the Clarivate Analytics Web of Science Core Collection databases by an experienced information specialist (JF). The search strategy included strings of terms and synonyms to reflect two concepts:

Concept 1: Carbon pricing. This included the three most important greenhouse gases (carbon dioxide, methane, nitrous oxide)

Concept 2: Health. This included general health terms as well as socioeconomic and health risk factors

The searches were limited to those published in 2010 or later and published in English or Spanish. No other filters or limits were added.

The search strategy was refined with the project team until the retrieved results reflected the scope of the project and contained relevant papers already known to the team. The agreed draft searches were adapted for each database to incorporate database-specific syntax and controlled vocabularies. The Full details of the search strings used for each database can be found in the appendix.

# Databases

Databases were searched on 23 June 2021. Results from the OvidSP databases Medline ALL, Embase, Global Health and Econlit were updated on 07 August 2023. Finally, all database results were updated on 20 October 2023.

Dates of coverage of the databases searched on 20 October 2023 are:

- OvidSP Medline ALL, 1946 to 19 October 2023.
- OvidSP Embase, 1974 to 19 October 2023.
- OvidSP Global Health, 1910 to Week 42 2023.
- OvidSP Econlit, 1886 to 12 October 2023.
- Clarivate Analytics Web of Science Core Collection, Data last updated 16 October 2023.
  This collection contains the following databases which were searched simultaneously:
  - Science Citation Index-Expanded, 1970-present;
  - Social Sciences Citation Index, 1970-present;
  - Arts & Humanities Citation Index, 1975-present;
  - Conference Proceedings Citation Index-Science, 1990-present;
  - Conference Proceedings Citation Index-Social Science & Humanities, 1990-present;
  - Emerging Sources Citation Index, 2015-present.
- Clarivate Analytics Web of Science Korean Journal Database, 1980-present. Data last updated 29 September 2023.
- Clarivate Analytics Web of Science SciELO Citation Index, 2002-present. Data last updated 14 October 2023.
- EBSCOhost Africa-Wide Information, complete database.
- EBSCOhost GreenFILE, complete database.

## Information management and deduplication

All citations identified by our searches were imported into EndNote 21 software. Duplicates were identified and removed using the method described on the London School of Hygiene & Tropical Medicine Library & Archives Service blog.^1^ Results were compared against previously entered references to remove duplicates. The order of duplication is outlined below.

| **2021** | **August 2023** | **October 2023** |
| --- | --- | --- |
| Medline | 2021 duplicated results | 2021 duplicated results |
| Embase | Medline | August 2023 duplicated results |
| Global Health | Embase | Medline |
| Econlit | Global Health | Embase |
| Africa-Wide Information | Econlit | Global Health |
| GreenFILE | Africa-Wide Information | Econlit |
| Web of Science Core Collection | GreenFILE | Africa-Wide Information |
| Korean Journal Database | Web of Science Core Collection | GreenFILE |
| SciELO | Korean Journal Database | Web of Science Core Collection |
|  | SciELO | Korean Journal Database |
|  |  | SciELO |

# Results

Number of results pre-and post-deduplication are listed in the tables below. A total of 5924 results were forwarded to the project team for screening.

| Database name | Total number of results retrieved on 23 June 2021 | Total number of results retrieved on 07 August 2023 | Total number of results retrieved on 20 October 2023 |
| --- | --- | --- | --- |
| Medline | 771 | 306 | 1056 |
| Embase | 1335 | 615 | 1386 |
| Global Health | 448 | 189 | 662 |
| Econlit | 516 | 154 | 667 |
| Africa-Wide Information | 82 | N/A | 94 |
| GreenFILE | 487 | N/A | 645 |
| Web of Science Core Collection databases (all searched simultaneously) | 2269 | N/A | 3274 |
| Korean Journal Database | 14 | N/A | 16 |
| SciELO Citation Index | 11 | N/A | 4 |
| **Total** | **5933** | **1264** | **7804** |

| Database name | Total number of results after duplicates removed 23 June 2021 | Total number of results after duplicates removed 07 August 2023 | Total number of results after duplicates removed 20 October 2023 |
| --- | --- | --- | --- |
| Medline | 715 | 881^[[1]](#footnote-1)^ | 17 |
| Embase | 931 |  | 25 |
| Global Health | 290 |  | 34 |
| Econlit | 487 |  | 14 |
| Africa-Wide Information | 37 | N/A | 2 |
| GreenFILE | 299 | N/A | 85 |
| Web of Science Core Collection databases (all searched simultaneously) | 1362 | N/A | 729 |
| Korean Journal Database | 10 | N/A | 1 |
| SciELO Citation Index | 9 | N/A | 17 |
| **Total** | **4132** | **881** | **911** |

# Appendix References

1. Falconer J. Removing duplicates from an EndNote Library. Library & Archives Service Blog [Internet]: London School of Hygiene & Tropical Medicine. 2018. [cited 2020]. Available from: <https://blogs.lshtm.ac.uk/library/2018/12/07/removing-duplicates-from-an-endnote-library/>.

# Appendix: Search strategies

This appendix provides full details of all search strings used for bibliographic databases, with dates and number of references returned and notes explaining any unusual search techniques or syntax. The EndNote 21import order is provided, as the deduplication technique keeps the first uploaded copy of the reference by default.

Searches run on 20 October 2023 are included below.

## Medline ALL

| Database name | Medline ALL |
| --- | --- |
| Database platform | OvidSP |
| Dates of database coverage | 1946 to 19 October 2023 |
| Date searched | 20 October 2023 |
| Searched by | JF |
| Number of results | 1056 |
| EndNote import order | 1 |
| Search strategy notes | Search lines ending in a ‘/’ are subject heading searches. Search lines beginning ‘exp’ are exploded subject heading searches. Two-letter codes at the end of search lines designate the fields to search. Fields codes used are: TI: title AB: abstract adj*n* searches for words within *n* words of each other. or/*x-y* combines search sets in the range *x-y* with Boolean operator OR. * is used for truncation of words. |

| # | Search terms | Results |
| --- | --- | --- |
| 1 | climate change/ or global warming/ | 30628 |
| 2 | Greenhouse Gases/ or Greenhouse Effect/ | 8129 |
| 3 | Carbon Dioxide/ or carbon/ | 166102 |
| 4 | Methane/ | 22402 |
| 5 | Nitrous Oxide/ | 15774 |
| 6 | exp fossil fuels/ or exp particulate matter/ | 107129 |
| 7 | Vehicle Emissions/ | 11891 |
| 8 | or/1-7 | 334556 |
| 9 | "Costs and Cost Analysis"/ | 51558 |
| 10 | Taxes/ | 7611 |
| 11 | Commerce/ | 29378 |
| 12 | Financing, Government/ | 21386 |
| 13 | or/9-12 | 106443 |
| 14 | 8 and 13 | 1626 |
| 15 | ((greenhouse gas* or ghg or carbon or fuel* or energy or particulate* or decarbon* or climate or co2 or ch4 or methane or n2o or nitro* oxide* or emission*) adj3 (price* or prici* or tax or taxes or taxation or trade* or trading or credit* or fiscal or subsid*)).ti,ab. | 3869 |
| 16 | ("cap and trade" or "cap and invest").ti,ab. | 128 |
| 17 | or/14-16 | 5199 |
| 18 | exp Health/ | 440531 |
| 19 | "Quality of Life"/ | 273704 |
| 20 | exp morbidity/ or exp mortality/ | 1034380 |
| 21 | Disease/ | 69267 |
| 22 | quality-adjusted life years/ | 15851 |
| 23 | "cost of illness"/ | 31755 |
| 24 | exp Exercise/ | 248984 |
| 25 | exp Diet/ | 331156 |
| 26 | cold-shock response/ or exp heat-shock response/ | 11996 |
| 27 | Sedentary Behavior/ | 13714 |
| 28 | Noise/ | 23008 |
| 29 | Crowding/ | 3813 |
| 30 | Air Pollution/ | 40348 |
| 31 | Accidents, Traffic/ | 48518 |
| 32 | exp Food Supply/ | 17357 |
| 33 | ((health* or well-being or wellbeing or morbidity or mortality or disease* or illness* or DALY* or life year* or burden of disease* or QALY* or death* or (life adj2 satisf*) or wellness or "quality of life" or QOL) not "soil").ti,ab. | 8819466 |
| 34 | (physical exercise or physical activity or diet* or nutrition* or (thermal adj1 (comfort or stress)) or (exposure adj2 (cold or heat or temperature*)) or walk* or sedentary or noise or noisy or crowding or overcrowding or air-quality or clean air or traffic accident*).ti,ab. | 1455967 |
| 35 | (energy poverty or food poverty or food security or food insecurity).ti,ab. | 18177 |
| 36 | (climate adj4 (benefit* or co-benefit*)).ti,ab. | 685 |
| 37 | or/18-36 | 10519151 |
| 38 | 17 and 37 | 1426 |
| 39 | limit 38 to yr="2010 -Current" | 1073 |
| 40 | limit 39 to (english or spanish) | 1060 |
| 41 | remove duplicates from 40 | 1056 |

## Embase

| Database name | Embase |
| --- | --- |
| Database platform | OvidSP |
| Dates of database coverage | 1974 to 19 October 2023 |
| Date searched | 20 October 2023 |
| Searched by | JF |
| Number of results | 1386 |
| EndNote import order | 2 |
| Search strategy notes | Search lines ending in a ‘/’ are subject heading searches. Search lines beginning ‘exp’ are exploded subject heading searches. Two-letter codes at the end of search lines designate the fields to search. Fields codes used are: TI: title AB: abstract adj*n* searches for words within *n* words of each other. or/*x-y* combines search sets in the range *x-y* with Boolean operator OR. * is used for truncation of words. |

| # | Search terms | Results |
| --- | --- | --- |
| 1 | exp climate change/ or climate resilience/ or greenhouse effect/ or greenhouse gas/ or carbon footprint/ | 84847 |
| 2 | carbon/ | 160013 |
| 3 | carbon dioxide/ | 124126 |
| 4 | methane/ | 35655 |
| 5 | nitrous oxide/ | 36383 |
| 6 | charcoal/ or coal/ or coke/ or diesel fuel/ or fossil fuel/ or fuel oil/ or gasoline/ or kerosene/ or liquefied natural gas/ or liquefied petroleum gas/ or natural gas/ or petroleum/ | 66700 |
| 7 | exp atmospheric particulate matter/ | 13058 |
| 8 | nitrous oxide emission/ | 1657 |
| 9 | exhaust gas/ | 21630 |
| 10 | or/1-9 | 480155 |
| 11 | tax/ | 16291 |
| 12 | commercial phenomena/ or market/ | 68531 |
| 13 | public expenditure/ | 431 |
| 14 | "cost"/ | 63371 |
| 15 | or/11-14 | 145347 |
| 16 | 10 and 15 | 3671 |
| 17 | ((greenhouse gas* or ghg or carbon or fuel* or energy or particulate* or decarbon* or climate or co2 or ch4 or methane or n2o or nitro* oxide* or emission*) adj3 (price* or prici* or tax or taxes or taxation or trade* or trading or credit* or fiscal or subsid*)).ti,ab. | 4081 |
| 18 | ("cap and trade" or "cap and invest").ti,ab. | 143 |
| 19 | or/16-18 | 7309 |
| 20 | exp health/ | 897847 |
| 21 | wellbeing/ or physical well-being/ or psychological well-being/ | 118255 |
| 22 | exp morbidity/ | 436261 |
| 23 | exp mortality/ | 1382640 |
| 24 | diseases/ | 148680 |
| 25 | exp disease burden/ | 85639 |
| 26 | exp death/ | 821629 |
| 27 | exp "quality of life"/ | 658941 |
| 28 | exp exercise/ | 435432 |
| 29 | exp diet/ | 405934 |
| 30 | nutrition/ | 122902 |
| 31 | thermal exposure/ | 5675 |
| 32 | sedentary lifestyle/ | 20740 |
| 33 | exp noise/ | 144578 |
| 34 | "crowding (area)"/ | 5518 |
| 35 | exp air quality/ | 37384 |
| 36 | traffic accident/ | 70460 |
| 37 | food security/ | 9035 |
| 38 | ((health* or well-being or wellbeing or morbidity or mortality or disease* or illness* or DALY* or life year* or burden of disease* or QALY* or death* or (life adj2 satisf*) or wellness or "quality of life" or QOL) not "soil").ti,ab. | 11918222 |
| 39 | (physical exercise or physical activity or diet* or nutrition* or (thermal adj1 (comfort or stress)) or (exposure adj2 (cold or heat or temperature*)) or walk* or sedentary or noise or noisy or crowding or overcrowding or air-quality or clean air or traffic accident*).ti,ab. | 1829439 |
| 40 | (energy poverty or food poverty or food security or food insecurity).ti,ab. | 19549 |
| 41 | (climate adj4 (benefit* or co-benefit*)).ti,ab. | 685 |
| 42 | or/20-41 | 14023206 |
| 43 | 19 and 42 | 1756 |
| 44 | limit 43 to yr="2010 -Current" | 1415 |
| 45 | limit 44 to (english or spanish) | 1403 |
| 46 | remove duplicates from 45 | 1386 |

## Global Health

| Database name | Global Health |
| --- | --- |
| Database platform | OvidSP |
| Dates of database coverage | 1910 to week 42 2023 |
| Date searched | 20 October 2023 |
| Searched by | JF |
| Number of results | 662 |
| EndNote import order | 3 |
| Search strategy notes | Search lines ending in a ‘/’ are subject heading searches. Search lines beginning ‘exp’ are exploded subject heading searches. Two-letter codes at the end of search lines designate the fields to search. Fields codes used are: TI: title AB: abstract adj*n* searches for words within *n* words of each other. or/*x-y* combines search sets in the range *x-y* with Boolean operator OR. * is used for truncation of words. |

| # | Search terms | Results |
| --- | --- | --- |
| 1 | exp climate change/ or greenhouse gases/ or greenhouse effect/ or carbon footprint/ | 16034 |
| 2 | carbon dioxide/ or nitrogen oxides/ | 12037 |
| 3 | carbon/ | 13206 |
| 4 | methane/ | 4962 |
| 5 | coal/ or coke/ or fossil fuels/ or exp fuel oils/ or kerosene/ or liquid petroleum gas/ or natural gas/ | 8729 |
| 6 | particulate matter/ | 9942 |
| 7 | exp emissions/ | 8385 |
| 8 | or/1-7 | 60716 |
| 9 | exp prices/ | 8975 |
| 10 | taxes/ or direct taxation/ or indirect taxation/ or levies/ or tax credits/ or tax incentives/ | 2310 |
| 11 | exp trade/ | 5626 |
| 12 | exp credit/ | 369 |
| 13 | exp subsidies/ | 844 |
| 14 | public expenditure/ | 248 |
| 15 | or/9-14 | 16994 |
| 16 | 8 and 15 | 585 |
| 17 | ((greenhouse gas* or ghg or carbon or fuel* or energy or particulate* or decarbon* or climate or co2 or ch4 or methane or n2o or nitro* oxide* or emission*) adj3 (price* or prici* or tax or taxes or taxation or trade* or trading or credit* or fiscal or subsid*)).ti,ab. | 829 |
| 18 | ("cap and trade" or "cap and invest").ti,ab. | 22 |
| 19 | or/16-18 | 1307 |
| 20 | exp health/ | 476584 |
| 21 | illness/ | 2932 |
| 22 | morbidity/ | 43470 |
| 23 | exp mortality/ | 203461 |
| 24 | exp diseases/ | 2982416 |
| 25 | "quality of life"/ | 30140 |
| 26 | exp food security/ or food access/ | 18289 |
| 27 | physical activity/ or exp exercise/ | 86690 |
| 28 | diet/ | 73510 |
| 29 | exp nutrition/ | 109793 |
| 30 | exposure/ | 83456 |
| 31 | noise/ or noise pollution/ | 3720 |
| 32 | exp crowding/ | 688 |
| 33 | air quality/ | 13227 |
| 34 | traffic accidents/ | 5084 |
| 35 | ((health* or well-being or wellbeing or morbidity or mortality or disease* or illness* or DALY* or life year* or burden of disease* or QALY* or death* or (life adj2 satisf*) or wellness or "quality of life" or QOL) not "soil").ti,ab. | 1988955 |
| 36 | (physical exercise or physical activity or diet* or nutrition* or (thermal adj1 (comfort or stress)) or (exposure adj2 (cold or heat or temperature*)) or walk* or sedentary or noise or noisy or crowding or overcrowding or air-quality or clean air or traffic accident*).ti,ab. | 706359 |
| 37 | (energy poverty or food poverty or food security or food insecurity).ti,ab. | 18402 |
| 38 | (climate adj4 (benefit* or co-benefit*)).ti,ab. | 245 |
| 39 | or/20-38 | 3833831 |
| 40 | 19 and 39 | 847 |
| 41 | limit 40 to yr="2010 -Current" | 704 |
| 42 | limit 41 to (english or spanish) | 664 |
| 43 | remove duplicates from 42 | 662 |

## Econlit

| Database name | Econlit |
| --- | --- |
| Database platform | OvidSP |
| Dates of database coverage | 1886 to 12 October 2023 |
| Date searched | 20 October 2023 |
| Searched by | JF |
| Number of results | 667 |
| EndNote import order | 4 |
| Search strategy notes | Two-letter codes at the end of search lines designate the fields to search. Fields codes used are: TI: title AB: abstract adj*n* searches for words within *n* words of each other. or/*x-y* combines search sets in the range *x-y* with Boolean operator OR. * is used for truncation of words. |

| # | Search terms | Results |
| --- | --- | --- |
| 1 | ((greenhouse gas* or ghg or carbon or fuel* or energy or particulate* or decarbon* or climate or co2 or ch4 or methane or n2o or nitro* oxide* or emission*) adj3 (price* or prici* or tax or taxes or taxation or trade* or trading or credit* or fiscal or subsid*)).ti,ab. | 14710 |
| 2 | ("cap and trade" or "cap and invest").ti,ab. | 870 |
| 3 | 1 or 2 | 14987 |
| 4 | ((health* or well-being or wellbeing or morbidity or mortality or disease* or illness* or DALY* or life year* or burden of disease* or QALY* or death* or (life adj2 satisf*) or wellness or "quality of life" or QOL) not "soil").ti,ab. | 96701 |
| 5 | (physical exercise or physical activity or diet* or nutrition* or (thermal adj1 (comfort or stress)) or (exposure adj2 (cold or heat or temperature*)) or walk* or sedentary or noise or noisy or crowding or overcrowding or air-quality or clean air or traffic accident*).ti,ab. | 24474 |
| 6 | (energy poverty or food poverty or food security or food insecurity).ti,ab. | 5035 |
| 7 | (climate adj4 (benefit* or co-benefit*)).ti,ab. | 418 |
| 8 | or/4-7 | 119977 |
| 9 | 3 and 8 | 917 |
| 10 | limit 9 to yr="2010 -Current" | 688 |
| 11 | limit 10 to (english or spanish) | 667 |

## Africa-Wide Information

| Database name | Africa-Wide Information |
| --- | --- |
| Database platform | EBSCOhost |
| Dates of database coverage | Complete database as of search date |
| Date searched | 20 October 2023 |
| Searched by | JF |
| Number of results | 94 |
| EndNote import order | 5 |
| Search strategy notes | Two-letter codes at the beginning of search lines designate the fields to search. Fields codes used are: TI: title AB: abstract * is used for truncation of words. N*n* searches for terms within *n* words of each other. |

| # | Search terms | Results |
| --- | --- | --- |
| S1 | (TI (("greenhouse gas*" or ghg or carbon or fuel* or energy or particulate* or decarbon* or climate or "co2" or "ch4" or methane or "n2o" or "nitro* oxide*" or emission*) N3 (price* or prici* or tax or taxes or taxation or trade* OR trading or credit* or fiscal or subsid*))) or (AB (("greenhouse gas*" or ghg or carbon or fuel* or energy or particulate* or decarbon* or climate or "co2" or "ch4" or methane or "n2o" or "nitro* oxide*" or emission*) N3 (price* or prici* or tax or taxes or taxation or trade* OR trading or credit* or fiscal or subsid*))) | 2,204 |
| S2 | (TI ("cap and trade" or "cap and invest")) or (AB ("cap and trade" or "cap and invest")) | 8 |
| S3 | S1 OR S2 | 2,205 |
| S4 | (TI ((health* or "well-being" or wellbeing or morbidity or mortality or disease* OR illness* OR DALY* OR "life year*" OR "burden of disease*" OR QALY* OR death* OR (life N2 satisf*) OR wellness OR "quality of life" OR QOL) not "soil")) or (AB ((health* or "well-being" or wellbeing or morbidity or mortality or disease* OR illness* OR DALY* OR "life year*" OR "burden of disease*" OR QALY* OR death* OR (life N2 satisf*) OR wellness OR "quality of life" OR QOL) not "soil")) | 582,704 |
| S5 | (TI ("physical exercise" or "physical activity" or diet* or nutrition* or (thermal n1 (comfort or stress)) or (exposure n2 (cold or heat or temperature*) ) or walk* or sedentary or noise or noisy or crowding or overcrowding or "air-quality" or "clean air" or "traffic accident*")) or (AB ("physical exercise" or "physical activity" or diet* or nutrition* or (thermal n1 (comfort or stress)) or (exposure n2 (cold or heat or temperature*) ) or walk* or sedentary or noise or noisy or crowding or overcrowding or "air-quality" or "clean air" or "traffic accident*")) | 94,776 |
| S6 | (TI ("energy poverty" or "food poverty" or "food security" or "food insecurity")) or (AB ("energy poverty" or "food poverty" or "food security" or "food insecurity")) | 9,133 |
| S7 | (TI (climate n4 (benefit or "co-benefit"))) or (AB (climate n4 (benefit or "co-benefit"))) | 55 |
| S8 | S4 OR S5 OR S6 OR S7 | 647,945 |
| S9 | S3 AND S8 | 170 |
| S10 | S9 Limiters - Year Published: 2010-2023 | 97 |
| S11 | S10 Limiters - Language: English, Spanish | 94 |

## GreenFILE

| Database name | GreenFILE |
| --- | --- |
| Database platform | EBSCOhost |
| Dates of database coverage | Complete database as of search date |
| Date searched | 20 October 2023 |
| Searched by | JF |
| Number of results | 645 |
| EndNote import order | 6 |
| Search strategy notes | Two-letter codes at the beginning of search lines designate the fields to search. Fields codes used are: TI: title AB: abstract * is used for truncation of words. N*n* searches for terms within *n* words of each other. |

| # | Query | Results |
| --- | --- | --- |
| S1 | (TI (("greenhouse gas*" or ghg or carbon or fuel* or energy or particulate* or decarbon* or climate or "co2" or "ch4" or methane or "n2o" or "nitro* oxide*" or emission*) N3 (price* or prici* or tax or taxes or taxation or trade* OR trading or credit* or fiscal or subsid*))) or (AB (("greenhouse gas*" or ghg or carbon or fuel* or energy or particulate* or decarbon* or climate or "co2" or "ch4" or methane or "n2o" or "nitro* oxide*" or emission*) N3 (price* or prici* or tax or taxes or taxation or trade* OR trading or credit* or fiscal or subsid*))) | 13,647 |
| S2 | (TI ("cap and trade" or "cap and invest")) or (AB ("cap and trade" or "cap and invest")) | 1,239 |
| S3 | S1 OR S2 | 14,083 |
| S4 | (TI ((health* or "well-being" or wellbeing or morbidity or mortality or disease* OR illness* OR DALY* OR "life year*" OR "burden of disease*" OR QALY* OR death* OR (life N2 satisf*) OR wellness OR "quality of life" OR QOL) not "soil")) or (AB ((health* or "well-being" or wellbeing or morbidity or mortality or disease* OR illness* OR DALY* OR "life year*" OR "burden of disease*" OR QALY* OR death* OR (life N2 satisf*) OR wellness OR "quality of life" OR QOL) not "soil")) | 116,508 |
| S5 | (TI ("physical exercise" or "physical activity" or diet* or nutrition* or (thermal n1 (comfort or stress)) or (exposure n2 (cold or heat or temperature*) ) or walk* or sedentary or noise or noisy or crowding or overcrowding or "air-quality" or "clean air" or "traffic accident*")) or (AB ("physical exercise" or "physical activity" or diet* or nutrition* or (thermal n1 (comfort or stress)) or (exposure n2 (cold or heat or temperature*) ) or walk* or sedentary or noise or noisy or crowding or overcrowding or "air-quality" or "clean air" or "traffic accident*")) | 58,387 |
| S6 | (TI ("energy poverty" or "food poverty" or "food security" or "food insecurity")) or (AB ("energy poverty" or "food poverty" or "food security" or "food insecurity")) | 3,962 |
| S7 | (TI (climate n4 (benefit or "co-benefit"))) or (AB (climate n4 (benefit or "co-benefit"))) | 1,109 |
| S8 | S4 OR S5 OR S6 OR S7 | 163,883 |
| S9 | S3 AND S8 | 1,007 |
| S10 | S9 Limiters - Publication Date: 20100101-20231231 | 645 |

## Web of Science Core Collection

| Database name | Web of Science Core Collection. This contains the following databases which are all searched together:   - Science Citation Index Expanded (SCI-Expanded) - Social Sciences Citation Index (SSCI) - Arts & Humanities Citation Index (A&HCI) - Conference Proceedings Citation Index – Science (CPCI-S) - Conference Proceedings Citation Index - Social Science & Humanities (CPCI-SSH) - Emerging Sources Citation Index (ESCI) |
| --- | --- |
| Database platform | Clarivate Analytics Web of Science |
| Dates of database coverage | SCI-Expanded, 1970-present SSCI, 1970-present A&HCI, 1975-present CPCI-S, 1990-present CPCI-SSH, 1990-present ESCI, 2015-present  Data last updated: 2023-10-16 |
| Date searched | 20 October 2023 |
| Searched by | JF |
| Number of results | 3274 |
| EndNote import order | 7 |
| Search strategy notes | * is used for truncation.  Two-letter codes at the beginning of search lines designate the fields to search. Fields codes used are: TI: title AB: abstract  NEAR/*n* searches for words within *n* words of each other. |

| # | Search terms | Results |
| --- | --- | --- |
| 1 | TI=(("greenhouse gas*" or "ghg" or "carbon" or "fuel*" or "energy" or "particulate*" or "decarbon*" or "climate" or "co2" or "ch4" or "methane" or "n2o" or "nitro* oxide*" or "emission*") NEAR/3 ("price*" or "prici*" or "tax" or "taxes" or "taxation" or "trade*" OR "trading" or "credit*" or "fiscal" or "subsid*") ) or AB=(("greenhouse gas*" or "ghg" or "carbon" or "fuel*" or "energy" or "particulate*" or "decarbon*" or "climate" or "co2" or "ch4" or "methane" or "n2o" or "nitro* oxide*" or "emission*") NEAR/3 ("price*" or "prici*" or "tax" or "taxes" or "taxation" or "trade*" OR "trading" or "credit*" or "fiscal" or "subsid*") ) | 55225 |
| 2 | TI=("cap and trade" or "cap and invest") or AB=("cap and trade" or "cap and invest") | 1560 |
| 3 | #2 OR #1 | 55694 |
| 4 | TI=(("health*" or "well-being" or "wellbeing" or "morbidity" or "mortality" or "disease*" OR "illness*" OR "DALY*" OR "life year*" OR "burden of disease*" OR "QALY*" OR "death*" OR ("life" NEAR/2 "satisf*") OR "wellness" OR "quality of life" OR "QOL") not "soil") or AB=(("health*" or "well-being" or "wellbeing" or "morbidity" or "mortality" or "disease*" OR "illness*" OR "DALY*" OR "life year*" OR "burden of disease*" OR "QALY*" OR "death*" OR ("life" NEAR/2 "satisf*") OR "wellness" OR "quality of life" OR "QOL") not "soil") | 9026751 |
| 5 | (TI=("physical exercise" or "physical activity" or "diet*" or "nutrition*" or ("thermal" near/1 ("comfort" or "stress") ) or ("exposure" near/2 ("cold" or "heat" or "temperature*") ) or walk* or "sedentary" or "noise" or "noisy" or "crowding" or "overcrowding" or "air-quality" or "clean air" or "traffic accident*") or AB=("physical exercise" or "physical activity" or "diet*" or "nutrition*" or ("thermal" near/1 ("comfort" or "stress") ) or ("exposure" near/2 ("cold" or "heat" or "temperature*") ) or walk* or "sedentary" or "noise" or "noisy" or "crowding" or "overcrowding" or "air-quality" or "clean air" or "traffic accident*")) | 2560262 |
| 6 | TI=("energy poverty" or "food poverty" or "food security" or "food insecurity") or AB=("energy poverty" or "food poverty" or "food security" or "food insecurity") | 45750 |
| 7 | TI=("climate" near/4 ("benefit" or "co-benefit*") ) or AB=("climate" near/4 ("benefit" or "co-benefit*") ) | 1549 |
| 8 | #7 OR #6 OR #5 OR #4 | 10994253 |
| 9 | #8 AND #3 | 3822 |
| 10 | #8 AND #3 and English or Spanish (Languages) | 3778 |
| 11 | #10 Timespan: 2010-01-01 to 2023-12-31 | 3274 |

## Korean Journal Database

| Database name | KCI-Korean Journal Database |
| --- | --- |
| Database platform | Clarivate Analytics Web of Science |
| Dates of database coverage | 1980-present  Data last updated 2023-09-29 |
| Date searched | 20 October 2023 |
| Searched by | JF |
| Number of results | 16 |
| EndNote import order | 9 |
| Search strategy notes | * is used for truncation.  Two-letter codes at the beginning of search lines designate the fields to search. Fields codes used are: TI: title AB: abstract  NEAR/*n* searches for words within *n* words of each other. |

| # | Search terms | Results |
| --- | --- | --- |
| 1 | TI=(("greenhouse gas*" or "ghg" or "carbon" or "fuel*" or "energy" or "particulate*" or "decarbon*" or "climate" or "co2" or "ch4" or "methane" or "n2o" or "nitro* oxide*" or "emission*") NEAR/3 ("price*" or "prici*" or "tax" or "taxes" or "taxation" or "trade*" OR "trading" or "credit*" or "fiscal" or "subsid*") ) or AB=(("greenhouse gas*" or "ghg" or "carbon" or "fuel*" or "energy" or "particulate*" or "decarbon*" or "climate" or "co2" or "ch4" or "methane" or "n2o" or "nitro* oxide*" or "emission*") NEAR/3 ("price*" or "prici*" or "tax" or "taxes" or "taxation" or "trade*" OR "trading" or "credit*" or "fiscal" or "subsid*") ) | 1954 |
| 2 | TI=("cap and trade" or "cap and invest") or AB=("cap and trade" or "cap and invest") | 60 |
| 3 | #2 OR #1 | 1968 |
| 4 | TI=(("health*" or "well-being" or "wellbeing" or "morbidity" or "mortality" or "disease*" OR "illness*" OR "DALY*" OR "life year*" OR "burden of disease*" OR "QALY*" OR "death*" OR ("life" NEAR/2 "satisf*") OR "wellness" OR "quality of life" OR "QOL") not "soil") or AB=(("health*" or "well-being" or "wellbeing" or "morbidity" or "mortality" or "disease*" OR "illness*" OR "DALY*" OR "life year*" OR "burden of disease*" OR "QALY*" OR "death*" OR ("life" NEAR/2 "satisf*") OR "wellness" OR "quality of life" OR "QOL") not "soil") | 213122 |
| 5 | (TI=("physical exercise" or "physical activity" or "diet*" or "nutrition*" or ("thermal" near/1 ("comfort" or "stress") ) or ("exposure" near/2 ("cold" or "heat" or "temperature*") ) or walk* or "sedentary" or "noise" or "noisy" or "crowding" or "overcrowding" or "air-quality" or "clean air" or "traffic accident*") or AB=("physical exercise" or "physical activity" or "diet*" or "nutrition*" or ("thermal" near/1 ("comfort" or "stress") ) or ("exposure" near/2 ("cold" or "heat" or "temperature*") ) or walk* or "sedentary" or "noise" or "noisy" or "crowding" or "overcrowding" or "air-quality" or "clean air" or "traffic accident*")) | 70883 |
| 6 | TI=("energy poverty" or "food poverty" or "food security" or "food insecurity") or AB=("energy poverty" or "food poverty" or "food security" or "food insecurity") | 563 |
| 7 | TI=("climate" near/4 ("benefit" or "co-benefit*") ) or AB=("climate" near/4 ("benefit" or "co-benefit*") ) | 12 |
| 8 | #7 OR #6 OR #5 OR #4 | 264301 |
| 9 | #8 AND #3 | 119 |
| 10 | #9 Timespan: 2010-01-01 to 2023-12-31 | 99 |
| 11 | #10 and English (Languages) | 16 |

## SciELO Citation Index

| Database name | SciELO Citation Index |
| --- | --- |
| Database platform | Clarivate Analytics Web of Science |
| Dates of database coverage | 2002-present  Data last updated 2023-10-14 |
| Date searched | 20 October 2023 |
| Searched by | JF |
| Number of results | 17 |
| EndNote import order | 8 |
| Search strategy notes | * is used for truncation.  Two-letter codes at the beginning of search lines designate the fields to search. Fields codes used are: TI: title AB: abstract  NEAR/*n* searches for words within *n* words of each other. |

| # | Search terms | Results |
| --- | --- | --- |
| 1 | TI=(("greenhouse gas*" or "ghg" or "carbon" or "fuel*" or "energy" or "particulate*" or "decarbon*" or "climate" or "co2" or "ch4" or "methane" or "n2o" or "nitro* oxide*" or "emission*") NEAR/3 ("price*" or "prici*" or "tax" or "taxes" or "taxation" or "trade*" OR "trading" or "credit*" or "fiscal" or "subsid*") ) or AB=(("greenhouse gas*" or "ghg" or "carbon" or "fuel*" or "energy" or "particulate*" or "decarbon*" or "climate" or "co2" or "ch4" or "methane" or "n2o" or "nitro* oxide*" or "emission*") NEAR/3 ("price*" or "prici*" or "tax" or "taxes" or "taxation" or "trade*" OR "trading" or "credit*" or "fiscal" or "subsid*") ) | 351 |
| 2 | TI=("cap and trade" or "cap and invest") or AB=("cap and trade" or "cap and invest") | 3 |
| 3 | #2 OR #1 | 352 |
| 4 | TI=(("health*" or "well-being" or "wellbeing" or "morbidity" or "mortality" or "disease*" OR "illness*" OR "DALY*" OR "life year*" OR "burden of disease*" OR "QALY*" OR "death*" OR ("life" NEAR/2 "satisf*") OR "wellness" OR "quality of life" OR "QOL") not "soil") or AB=(("health*" or "well-being" or "wellbeing" or "morbidity" or "mortality" or "disease*" OR "illness*" OR "DALY*" OR "life year*" OR "burden of disease*" OR "QALY*" OR "death*" OR ("life" NEAR/2 "satisf*") OR "wellness" OR "quality of life" OR "QOL") not "soil") | 224477 |
| 5 | (TI=("physical exercise" or "physical activity" or "diet*" or "nutrition*" or ("thermal" near/1 ("comfort" or "stress") ) or ("exposure" near/2 ("cold" or "heat" or "temperature*") ) or walk* or "sedentary" or "noise" or "noisy" or "crowding" or "overcrowding" or "air-quality" or "clean air" or "traffic accident*") or AB=("physical exercise" or "physical activity" or "diet*" or "nutrition*" or ("thermal" near/1 ("comfort" or "stress") ) or ("exposure" near/2 ("cold" or "heat" or "temperature*") ) or walk* or "sedentary" or "noise" or "noisy" or "crowding" or "overcrowding" or "air-quality" or "clean air" or "traffic accident*")) | 53425 |
| 6 | TI=("energy poverty" or "food poverty" or "food security" or "food insecurity") or AB=("energy poverty" or "food poverty" or "food security" or "food insecurity") | 1466 |
| 7 | TI=("climate" near/4 ("benefit" or "co-benefit*") ) or AB=("climate" near/4 ("benefit" or "co-benefit*") ) | 11 |
| 8 | #7 OR #6 OR #5 OR #4 | 257460 |
| 9 | #8 AND #3 | 28 |
| 10 | #9 Timespan: 2010-01-01 to 2023-12-31 | 24 |
| 11 | #10 and Spanish or English (Languages) | 17 |

1. Total number of results across all 4 databases once duplicates removed. [↑](#footnote-ref-1)
